# Copy number gain of ZEB1 mediates a double-negative feedback loop with miR-33a-5p that regulates EMT and bone metastasis of prostate cancer dependent on TGF-β signaling

**DOI:** 10.7150/thno.36735

**Published:** 2019-08-14

**Authors:** Yuhu Dai, Zhengquan Wu, Chuandong Lang, Xin Zhang, Shaofu He, Qing Yang, Wei Guo, Yingrong Lai, Hong Du, Xinsheng Peng, Dong Ren

**Affiliations:** 1 Department of Orthopaedic Surgery, the First Affiliated Hospital of Sun Yat-sen University, 510080, Guangzhou, China; 2 Guangdong Provincial Key Laboratory of Orthopedics and Traumatology, 510080,Guangzhou, Guangdong Province, China; 3 Clinical Experimental Center, Jiangmen Central Hospital, Affiliated Jiangmen Hospital of Sun Yat-sen University, Jiangmen, China; 4 Department of radiology, the First Affiliated Hospital of Sun Yat-sen University, 510080, Guangzhou, China; 5 Department of Pathology, the First Affiliated Hospital of Sun Yat-sen University, 510080, Guangzhou, China; 6 Department of Pathology, the First People's Hospital of Guangzhou City, 510180, Guangzhou, Guangdong Province, China

**Keywords:** miR-33a-5p, ZEB1, double negative feedback loop, bone metastasis, prostate cancer

## Abstract

**Background**: The reciprocal repressive loop between ZEB1 and miRNAs has been extensively reported to play an important role in tumor progression and metastasis of various human tumor types. The aim of this study was to elucidate the role and the underlying mechanism of the double-negative feedback loop between ZEB1and miR-33a-5p in bone metastasis of prostate cancer (PCa).

**Methods**: miR-33a-5p expression was examined in 40 bone metastatic and 165 non-bone metastatic PCa tissues by real-time PCR. Statistical analysis was performed to evaluate the clinical correlation between miR-33a-5p expression and clinicopathological characteristics, and overall and bone metastasis-free survival in PCa patients. The biological roles of miR-33a-5p in bone metastasis of PCa were investigated both by EMT and the Transwell assay *in vitro*, and by a mouse model of left cardiac ventricle inoculation *in vivo*. siRNA library, real-time PCR and chromatin immunoprecipitation (ChIP) were used to identify the underlying mechanism responsible for the decreased expression of miR-33a-5p in PCa. Bioinformatics analysis, Western blotting and luciferase reporter analysis were employed to examine the relationship between miR-33a-5p and its potential targets. Clinical correlation of miR-33a-5p with its targets was examined in human PCa tissues and primary PCa cells.

**Results**: miR-33a-5p expression was downregulated in PCa tissues with bone metastasis and bone-derived cells, and low expression of miR-33a-5p strongly and positively correlated with advanced clinicopathological characteristics, and shorter overall and bone metastasis-free survival in PCa patients. Upregulating miR-33a-5p inhibited, while silencing miR-33a-5p promoted EMT, invasion and migration of PCa cells. Importantly, upregulating miR-33a-5p significantly repressed bone metastasis of PC-3 cells *in vivo*. Our results further revealed that recurrent ZEB1 upregulation induced by copy number gains transcriptionally inhibited miR-33a-5p expression, contributing to the reduced expression of miR-33a-5p in bone metastatic PCa tissues. In turn, miR-33a-5p formed a double negative feedback loop with ZEB1 in target-independent manner, which was dependent on TGF-β signaling. Finally, the clinical negative correlations of miR-33a-5p with ZEB1 expression and TGF-β signaling activity were demonstrated in PCa tissues and primary PCa cells.

**Conclusion**: Our findings elucidated that copy number gains of ZEB1-triggered a TGF-β signaling-dependent miR-33a-5p-mediated negative feedback loop was highly relevant to the bone metastasis of PCa.

## Introduction

Epithelial-mesenchymal transition (EMT) is an embryonic programmed event that has been reported to be implicated in several biological processes 1, 2, in which the cell morphology is converted from the epithelial-like profiles to the mesenchymal phenotype concomitant with the detachment of cells from intercellular adhesion and enhanced motility 3. Numerous studies have demonstrated that aberrant activation of this embryonic program significantly contributes to the increased invasion and migration of cancer cells and promotes distant metastasis in a variety of cancers 4, 5. EMT is initiated by multiple stimuli or cellular signaling by triggering the expression of EMT-inducing transcriptional repressors 6-8, including ZEB1/2, SNAI1/2 and TWIST1/2 9. Of these, ZEB1, as a critical EMT activator, has considerable relevance to tumor metastasis and correlates with poor patient prognosis 10, 11.

ZEB1 has been reported to induce the development of EMT by reducing the expression of basement membrane components 10. Several studies have reported that ZEB1 transcriptionally downregulates cell polarity factors, which further promotes EMT and metastasis of cancer cells 12, 13. Furthermore, accumulating evidence has shown that ZEB1 contributes to EMT and cancer metastasis by repressing the expression of anti-metastatic microRNAs (miRNAs), such as the miR-200 family 14. miRNAs are a class small noncoding RNAs that function post-transcriptional repressive role by sequence-specific binding to the mRNA of their targets 15. Aberrant expression of miRNAs have been inherently correlated with metastasis and progression of cancers 16-22. By simultaneously targeting a variety of EMT-inducing factors, including ZEB1, these important anti-metastatic miRNAs, alone or cooperatively, are sufficient to suppress EMT and tumor metastasis 23-25. These literatures indicate that the double-negative feedback loop between miRNAs and ZEB1 function as a key regulatory axis in EMT and metastasis program. Indeed, it has been reported that ZEB1 directly suppressed transcription of microRNA-200 family members miR-141 and miR-200c, which in turn targeted ZEB1 expression, revealing the pivotal role of the reciprocal repressive loop between ZEB1 and miR-141 and miR-200c in EMT and invasion of pancreatic, colorectal and breast cancer cells 26.

As one of the originally identified miRNAs, miR-33a-5p has been widely demonstrated to function as a tumor suppressor in multiple cancer types 27-31. In PCa, decreased expression of miR-33a-5p has been reported in two independent studies 32, 33, further reinforcing the tumor-suppressive role of miR-33a-5p in cancer. However, the clinical significance and biological functions of miR-33a-5p in bone metastasis of PCa remain undefined.

Here, we reported that miR-33a-5p was downregulated in PCa tissues with bone metastasis, which significantly correlated with differentiation, serum PSA level and Gleason grade, and predicted shorter bone metastasis-free survival in PCa patients. Gain and loss of function experiments demonstrated that upregulating miR-33a-5p inhibited EMT, invasion and migration of PCa cells *in vitro*, and repressed bone metastasis ability of PCa cells *in vivo*. Conversely, silencing miR-33a-5p yielded an opposite effect. Our results further revealed that miR-33a-5p suppressed bone metastasis of PCa by inactivating TGF-β signaling via directly targeting transforming growth factor beta receptor 1 (TGFBRI)* in vitro* and *in vivo*. Taken together, our results indicate that recurrent ZEB1 upregulation induced by copy number gains mediating a double negative feedback loop between miR-33a-5p and ZEB1 dependent on TGF-β signaling plays an important role in EMT and bone metastasis of PCa.

## Methods

### Cell culture

Human PCa cell lines 22RV1, PC-3, VCaP, DU145, LNCaP and normal prostate epithelial cells RWPE-1 were obtained from the Shanghai Chinese Academy of Sciences cell bank (China). RWPE-1 cells were grown in defined keratinocyte-SFM (1×) (Invitrogen). PC-3, LNCaP and 22Rv1 cells were cultured in RPMI-1640 medium (Life Technologies, Carlsbad, CA, US) supplemented with penicillin G (100 U/ml), streptomycin (100 mg/ml) and 10% fetal bovine serum (FBS, Life Technologies). DU145 and VCaP cells were grown in Dulbecco's modified Eagle's medium (Invitrogen) supplemented with 10% FBS. The C4-2B cell line was purchased from the MD Anderson Cancer Center and maintained in T-medium (Invitrogen) supplemented with 10 % FBS. All cell lines were grown under a humidified atmosphere of 5 % CO2 at 37 °C.

### Plasmid, small interfering RNA and transfection

The target sequences were PCR-amplified from genomic DNA. The PCR amplification product was verified by electrophoresis through a 1% agarose gel, and the targeted band was selected for purification. The PCR product and the vector were digested with restriction enzymes overnight. The ligation reaction was carried out in a refrigerator at 4 ° C for about 16 h, then competent E. coli were transformed with dilutions of each of the ligation reactions. Small-scale cultures of E. coli clones were prepared by inoculating 2 ml of LB liquid medium. The bacterial solution was uniformly coated on an LB solid medium containing the appropriate antibiotic, and cultured at 37 ° C for 12-16 h. Subsequently, 100 ml of LB medium containing the appropriate antibiotic was inoculated with a single colony of transformed bacteria, and the culture was incubated overnight at 37°C with vigorous shaking. The culture was centrifuged and EndoFree Maxi Plasmid Kit (TIANGEN, Chinese) was used for purification of the recombinant plasmid. The human miR-33a-5p gene plasmid was cloned into a pMSCV-puro retroviral vector (Clontech, Japan). The 3'UTR regions of TGFBRI and ZEB1 were cloned into pmirGLO vectors (Promega, USA). The constitutively active mutant TGFBRI plasmid was cloned into pSin-EF2 vectors (Addgene, USA). The list of primers used in cloning reactions is presented in Table S1. Knockdown of endogenous ZEB1 was performed by cloning two short hairpin RNA (shRNA) oligonucleotides into the pLKO.1 vector (Addgene, USA). The sequences of the two separate shRNA fragments were: shRNA#1, GCTGTTGTTCTGCCAACAGTTCTCGAGAACTGTTGGCAGAACAACAGC and shRNA#2: GCAACAATACAAGAGGTTAAACTCGAGTTTAACCTCTTGTATTGTTGC (synthesized by Invitrogen). Retroviral production and infection were performed as previously described 34. The (CAGAC) 12/pGL3 TGF-β/Smad-responsive luciferase reporter plasmid and the control plasmids (Clontech, Japan) were used to quantitatively assess the transcriptional activity of TGF-β signaling components. anti-miR-33a-5p plasmid, small interfering RNA (siRNA) for the ZEB1 and 21 transcriptional factors-associated with miR-33a-5p expression, corresponding control siRNAs and antagomiR-33a-5p were synthesized and purified by RiboBio. Transfection of miRNA, siRNAs, and plasmids was performed as previously described 35.

### RNA extraction, reverse transcription, and real-time PCR

Real-time PCR was performed as described previously 7. The primers are provided in Table S2. Primers for U6 and miR-33a-5p were synthesized and purified by RiboBio (Guangzhou, China). U6 or glyceraldehyde-3-phosphate dehydrogenase (GAPDH) was used as the endogenous control. Relative fold expressions were calculated with the comparative threshold cycle (2^-ΔΔCt^) method as previously described 36. The DNA copy number variation for ZEB1 was evaluated using Real time PCR primer Hs05175551_cn through TaqMan® Copy Number Assays (Life Technologies™). Cases and controls were simultaneously amplified according to the manufacturer's instructions. Briefly, 5ng/μl of DNA from both PCa samples and normal reference were mixed with the TaqMan® Genotyping Mastermix (Life Technologies™) using the ViiA 7 Real Time PCR instrument (Applied Biosystems). The RNAse P gene (RPPH1) was used as the diploid reference (TaqMan Copy Number Reference Assay -Life Technologies™). PCR conditions were as follows: 95 °C initial denaturation step for 10 minutes, 40 cycles at 95 °C for 15 seconds and 60 °C for 1 minute. All reactions were performed in triplicate and a negative control was included in each batch. Copy number was analyzed by the Copy Caller Software (Life Technologies™), and only the samples that presented Ct cycles <33 and a Z-score value ≤ 2.65 were considered in the analyses. A > 2 fold increase in the copy number variation of ZEB1 in the PCa samples in comparison to the normal control was considered as “Gain” and the samples with copy number variation number ≤ 2 as “No Gain”.

### Patients and tumor tissues

A total of 205 archived PCa tissues, including 165 primary PCa tissues without or no bone metastasis and 40 primary PCa tissues with bone metastasis were obtained during surgery or needle biopsy at The First People's Hospital of Guangzhou City (Guangzhou, China) between January 2003 and October 2008. Patients were diagnosed based on clinical and pathological evidence, and the specimens were immediately snap-frozen and stored in liquid nitrogen tanks. For the use of these clinical materials for research purposes, prior patient' consents and approval from the Institutional Research Ethics Committee were obtained (approval-No. [2014]A-011). The clinicopathological features of the patients are summarized in Table S3. The median of miR-33a-5p expression in PCa tissues was used to stratify high and low expression of miR-33a-5p.

### High throughput data processing and visualization

The copy number variation profile and methylation array profile of PCa dataset were downloaded from The Cancer Genome Atlas (TCGA; https://tcga-data.nci.nih.gov/tcga/). For copy number variation profile, the Level 3 Copy Number Variation (CNV) dataset of PCa in SNP6.0 microarray was downloaded from TCGA, and analyzed by GISTIC2.0 software as described previously (all parameters as the default) 37. The CNV number of each corresponding sample was procured and the CNV number of Amplification and Gain groups was defined as “Gain” and the rest as “No Gain”. The results were analyzed using Excel 2010 and figures were depicted using GraphPad 5 software. For methylation array profile: the DNA methylation dataset of PCa in 450K Methylation microarray was downloaded from TCGA, and the β-value (representing methylation level) of the related genes from the Level 3 data of each sample was procured. The MIR33A-related probe numbers from the methylation microarray data of 49 paired PCa tissues were exported using Excel 2010 and GraphPad 5 software. The differential β-value between PCa tissues and the adjacent normal tissue (ANT) in the paired PCa tissues, as well as between primary PCa tissues with bone metastasis and primary PCa tissues without bone metastasis were calculated, and the heat map was depicted by MeV4.6 software.

### Gene Set Enrichment Analysis (GSEA)

The analysis procedure of GSEA was performed as follows: the miRNA dataset of PCa was first downloadeded from TCGA, and the expression values of the corresponding miRNAs from the Level 3 data were procured. The log2 value of each sample was analyzed using Excel 2010 and GraphPad 5. For statistical analysis of the miRNA expression levels of all PCa tissues, paired or unpaired t-test were performed using SPSS version 19.0 (SPSS Inc., Chicago, IL, USA). GSEA was performed with miRNA dataset of PCa from TCGA as Expression dataset. The high and low expression levels of miR-33a-5p were stratified by the medium expression level of miRNA in 498 PCa tissues. Gene set was performed by Molecular Signatures Database v5.2 (http://software.broadinstitute.org/gsea/msigdb) (all processing parameters as the default).

### Western blotting

Western blotting was performed as described previously 38. Antibodies against E-cadherin, vimentin, fibronectin, ZEB1, TGFBRI, pSMAD3 and SMAD3 were purchased from Cell Signaling Technology, p65 from Proteintech and p84 from Invitroge. The membranes were stripped and reprobed with an anti-α-tubulin antibody (Sigma-Aldrich, USA) as the loading control.

### Luciferase assay

Cells (4 ×10^4^) were seeded in triplicate in 24-well plates and cultured for 24 h, and the assay was performed as previously described 39. Cells were transfected with 250 ng (CAGAC) 12/pGL3 reporter luciferase plasmid, 100 ng pmirGLO-TGFBRI or ZEB1-3'UTR, luciferase plasmid, or 5 ng pRL-TK Renilla plasmid (Promega, USA) using Lipofectamine 3000 (Invitrogen, USA) according to the manufacturer's recommendations. Luciferase and Renilla signals were measured 36 h after transfection using a Dual Luciferase Reporter Assay Kit (Promega) according to the manufacturer's protocol.

### miRNA immunoprecipitation

Cells were co-transfected with HA-Ago2, followed by immunoprecipitation using anti-HA-antibody as previously described 40. Real-time PCR analysis of the IP material was performed to test the association of the mRNA of ZEB1 and TGFBRI with the RISC complex.

### Chromatin immunoprecipitation (ChIP) assay

ChIP assay was performed according to the manufacturer's instructions of ChIP assay kit (Millipore, catalog: 17-371). PCa cells with or without lentiviral-mediated ectopic ZEB1 expression were first fixed with 1% formaldehyde to covalently crosslink proteins to DNA and then chromatin was harvested from the cells. Crosslinked DNA was sheared to 200-1,000 base pairs in length with sonication and then subjected to an immunoprecipitation procedure with the anti-ZEB1 antibody (Sigma-Aldrich, USA). Finally, PCR was used to measure enrichment of DNA fragments in the putative ZEB1-binding sites in the MIR33A promoter using the specific primers (Table S4).

### Immunohistochemistry

The immunohistochemistry procedures were performed as previously described 41. The slides were incubated overnight at 4°C in a humidified chamber with the TGFBRI, ZEB1 and SMAD3 antibodies diluted 1:100 in PBS. Tumor cell proportion was scored as follows: 0 (no positive tumor cells); 1 (<10% positive tumor cells); 2 (10-35% positive tumor cells); 3 (35-70% positive tumor cells) and 4 (>70% positive tumor cells). Staining intensity was graded according to the following criteria: 0 (no staining); 1 (weak staining, light yellow); 2 (moderate staining, yellow brown) and 3 (strong staining, brown). Staining index (SI) was calculated as the product of staining intensity score and the proportion of positive tumor cells. Using this method of assessment, we evaluated TGFBRI, ZEB1 and SMAD3 expression in PCa samples by determining the SI, with scores of 0, 1, 2, 3, 4, 6, 8, 9 or 12. Score 6 was the median of all sample tissues SI. High and low expression of TGFBRI, ZEB1 or SMAD3 were stratified by the follow criteria: The SI score of ≥6 was used to define tumors with high expression of TGFBRI, ZEB1 or SMAD3, and SI≤4 as tumors with low expression of TGFBRI, ZEB1 or SMAD3.

### Invasion and migration assays

The invasion and migration assays were performed as described previously 42. The cell count was performed under a microscope (x100).

### Animal study

All mouse experiments were approved by The Institutional Animal Care and Use Committee of Sun Yat-sen University (approval-No. L102012018090R), and housed as previously described 43. For the bone metastasis study, the BALB/c-nu mice (5-6 weeks old, 18-20 g) were anesthetized and inoculated into the left cardiac ventricle with 1 × 10^5^ PC-3 cells in 100 μl of PBS. Bone metastases were monitored, scored and analyzed by bioluminescent imaging (BLI), X-ray and H&E staining as previously described 44.

### Statistical analysis

All values were presented as the mean ± standard deviation (SD). Significant differences were determined using the GraphPad 5.0 software (USA). Student's t-test was used to determine statistical differences between the two groups. The chi-square test was used to analyze the relationship between miR-33a-5p expression and clinicopathological characteristics. For multivariate Cox regression, only independent variables with P<0.1 in univariate Cox regression were incorporated into multivariate Cox regression analysis using Forward LR method. Since both the differentiation and Gleason grade reflect the differentiation of prostate cancer tissues (Interaction), we only select Gleason grade, a more commonly used variable in the setting of prostate cancer, in multivariate analysis. P < 0.05 was considered significant. All experiments were repeated three times.

## Results

### miR-33a-5p is downregulated in PCa tissues with bone metastasis

**Figure 1:**
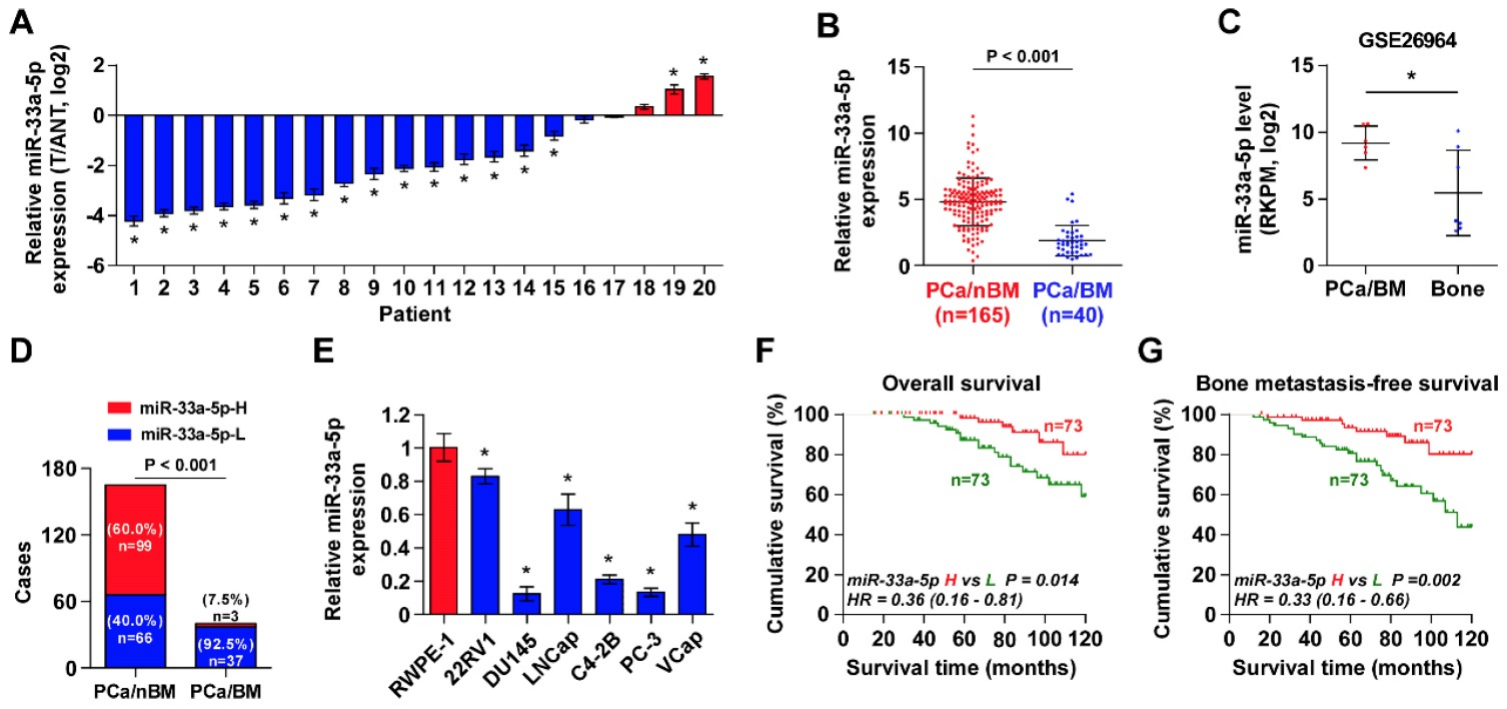
** miR-33a-5p is downregulated in PCa tissues with bone metastasis. A,** Real-time PCR analysis of miR-33a-5p expression in 20 paired PCa tissues and their matched adjacent normal tissues (ANT). Transcript levels were normalized to *U6* expression. *P < 0.05. **B,** Real-time PCR analysis of miR-33a-5p expression in primary PCa tissues without or no bone metastasis (PCa/nBM, n = 165) and primary PCa tissues with bone metastasis (PCa/BM, n = 40). Transcript levels were normalized to *U6* expression. *P < 0.05.** C,** miR-33a-5p expression in PCa/BM (n = 6) and bone metastatic tissues (Bone, n = 7) in our previous miRNA microarray dataset. **D,** Percentages and number of samples showed high or low miR-33a-5p expression in PCa/nBM and PCa/BM in our PCa tissues.** E,** Real-time PCR analysis of miR-33a-5p expression levels in normal prostate epithelial cell (RWPE-1), primary PCa cell 22RV1, brain metastatic cell line DU145, lymph node metastatic cell line LNCaP and three bone metastatic PCa cell lines (PC-3, C4-2B and VCaP). Transcript levels were normalized to *U6* expression. *P < 0.05.** F,** Kaplan-Meier analysis of overall survival curves of the PCa patients stratified by miR-33a-5p expression. **G,** Kaplan-Meier analysis of bone metastasis-free survival curves of the PCa patients stratified by miR-33a-5p expression.

To determine the clinical significance of miR-33a-5p in PCa, its expression was first examined in 20 paired PCa tissues and ANT by Real-time PCR. As shown in Figure 1A, the miR-33a-5p expression level in 15/20 PCa tissues (75%) was differentially downregulated compared with that in the ANT. Of note, miR-33a-5p expression was reduced in primary PCa tissues with bone metastasis (PCa/BM) compared with that in primary PCa tissues without bone metastasis (PCa/nBM) (Figure 1B), and further downregulated in bone metastatic tissues (Bone) in our previously reported miRNA microarray dataset (Figure 1C) 45. Moreover, the percentage of low expression of miR-33a-5p was higher in PCa/BM than that in PCa/nBM (Figure 1D). The miR-33a-5p expression was further examined in normal prostate epithelial cells RWPE-1 and six PCa cell lines. As shown in Figure 1E, miR-33a-5p expression was differentially downregulated in PCa cells compared with that in RWPE-1, especially in bone metastatic PCa cell lines PC-3 and C4-2B, and brain metastatic cell line DU145.

The correlation analysis of miR-33a-5p expression with clinicopathological characteristics in PCa patients revealed that miR-33a-5p expression inversely correlated with differentiation, serum PSA levels, Gleason grade and bone metastasis status in PCa patients (Table S5). More importantly, decreased expression of miR-33a-5p predicted poor overall and bone metastasis-free survival in PCa patients (Figure 1F and G). Univariate Cox regression analysis indicated that patients with decreased expression of miR-33a-5p had shorter overall survival (P = 0.014; hazard ratio = 0.36, 95% CI = 0.16 to 0.81) (Table S6) and bone metastasis-free survival (P = 0.002; hazard ratio = 0.33, 95% CI = 0.16 to 0.66) compared to patients with increased expression of miR-33a-5p (Table S7). Multivariate Cox regression analysis suggested that miR-33a-5p may be used as an independent factor for predicting poor overall survival (P = 0.020; hazard ratio = 0.33, 95% CI = 0.13 to 0.84) and bone metastasis-free survival (P = 0.021; hazard ratio = 0.35, 95% CI = 0.14 to 0.85) (Table S6 and 7). Taken together, these results indicated that reduced expression of miR-33a-5p might contribute to bone metastasis of PCa.

### Ectopic expression of miR-33a-5p represses bone metastasis of PCa *in vivo*

**Figure 2:**
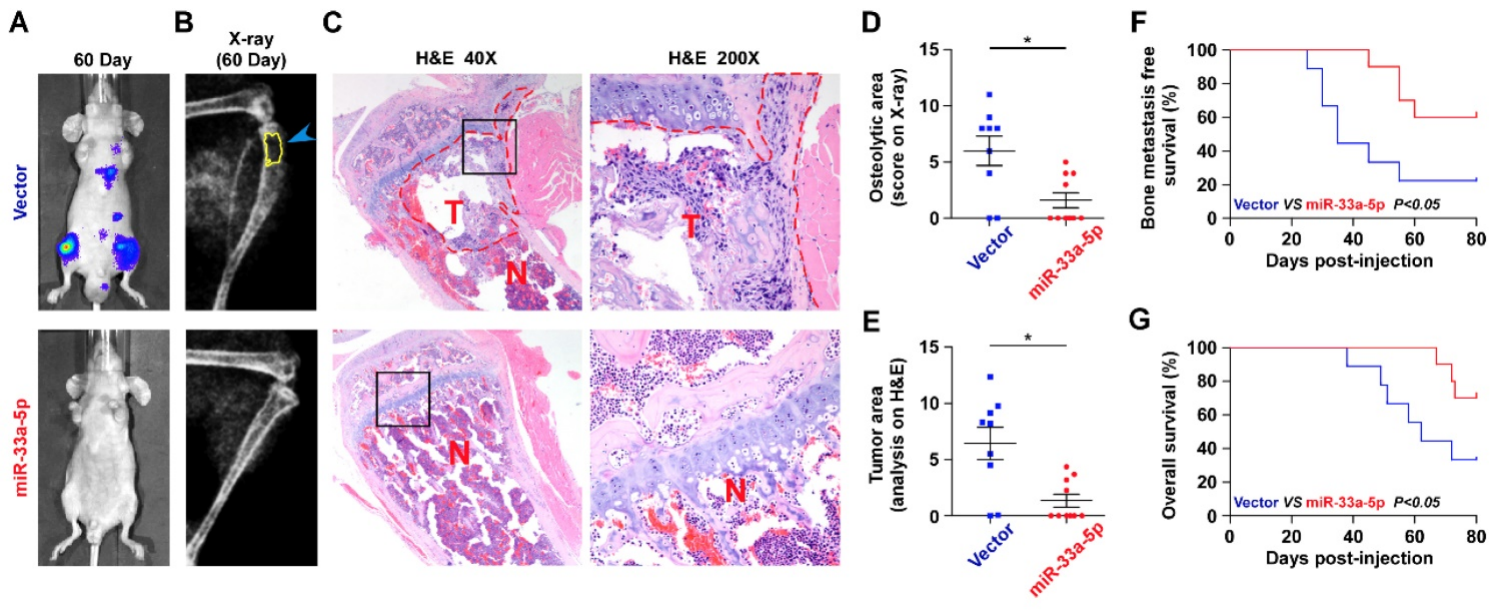
** Upregulating miR-33a-5p represses bone metastasis of PCa cells *in vivo*. A,** Representative BLIs signal of bone metastasis of a mouse from the indicated groups of mice on day 60.** B,** Representative radiographic images of bone metastases in the mice groups injected with vector and miR-33a-5p-overexpressing PC-3 cells respectively (arrows indicate osteolytic lesions).** C,** Representative H&E-stained sections of tibias from the indicated mouse (T, tumor; N, the adjacent non-tumor tissues). **D,** The sum of bone metastasis scores for each mouse in tumor-bearing mice inoculated with vector (n = 9) or miR-33a-5p-overexpressing (n = 10) cells. **E,** Histomorphometric analysis of bone osteolytic areas in the tibia of the indicated mice groups. *P < 0.05.** F and G.** Kaplan-Meier analysis of mouse bone metastasis-free **(F)** and overall **(G)** survival in the indicated mice groups.

The effect of miR-33a-5p on the bone metastasis of PCa *in vivo* was further investigated in a mouse model of bone metastasis, in which the luciferase-labeled vector or miR-33a-5p-stably overexpressing PC-3 cells were inoculated respectively into the left cardiac ventricle of male nude mice (Figure S1). The progression of bone metastasis was monitored by bioluminescence imaging (BLI) and X-ray analysis. As shown in Figure 2A-C, upregulation of miR-33a-5p markedly inhibited bone metastasis ability of PC-3 cells *in vivo* compared with the vector group by BLI and X-ray, as well as reduced the tumor burden in bone by H&E staining of the tumor sections from the tibias of injected mice. Furthermore, overexpression of miR-33a-5p reduced bone metastatic scores and osteolytic area of metastatic bone tumors, and prolonged bone metastasis-free and overall survival compared with the vector group (Figure 2D-E). These findings indicated that upregulating miR-33a-5p represses bone metastasis of PCa *in vivo*.

### Upregulation of miR-33a-5p inhibits EMT, invasion and migration

**Figure 3:**
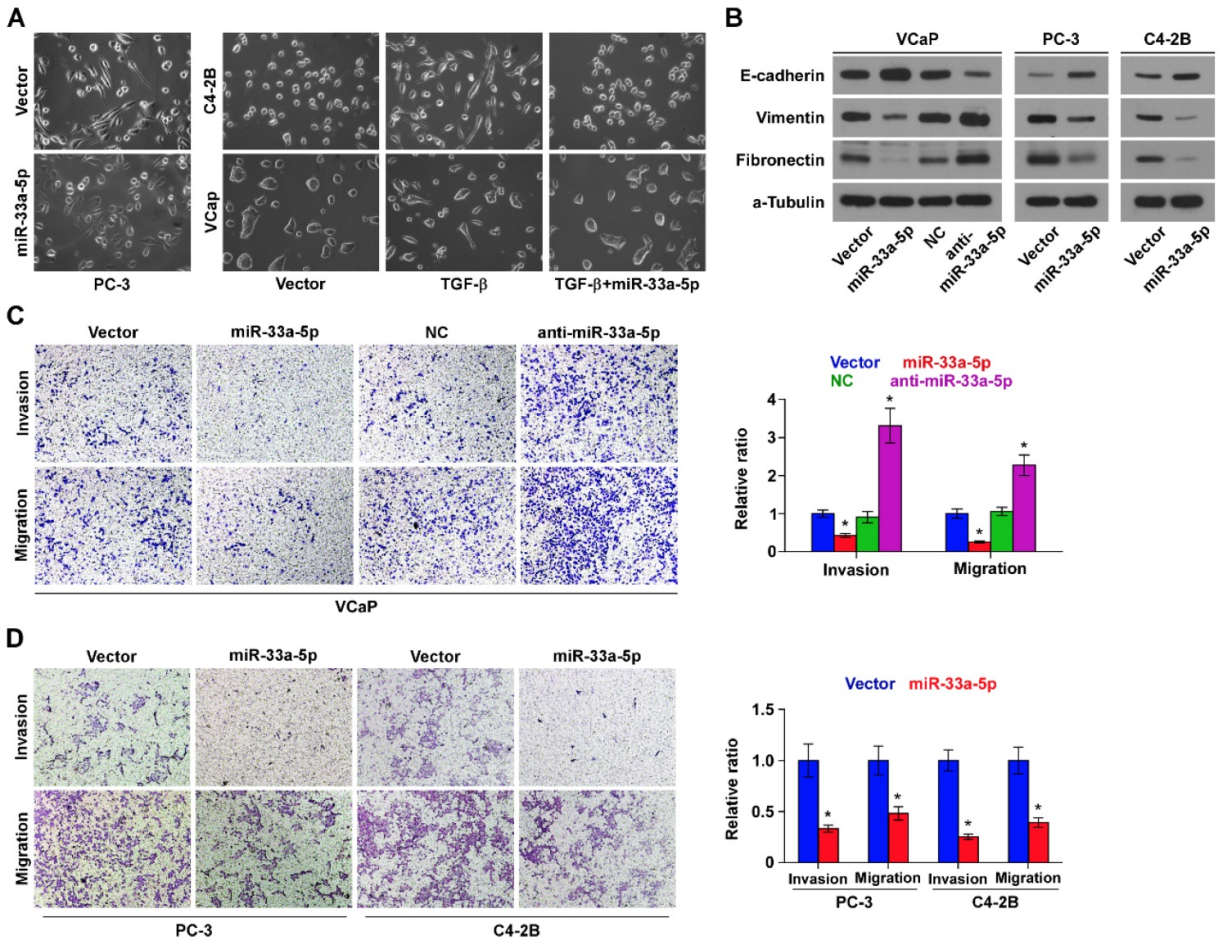
** Upregulating miR-33a-5p inhibits EMT, invasion and migration in PCa cells. A,** Upregulating miR-33a-5p converted a stick-like or long spindleshaped mesenchymal profile to a cobblestone-like or a short spindle-shaped epithelial morphology in PC-3 cells (left panel). Upregulating miR-33a-5p converted a stick-like or long spindleshaped mesenchymal profile to a cobblestone-like or a short spindle-shaped epithelial morphology in VCaP and C4-2B cells treated with TGF-β (5 ng/ml for 72h) (right panel). **B,** Western blot analysis of E-cadherin expression, Vimentin and Fibronectin in the indicated PCa cells. α-Tubulin served as the loading control. **C and D** Upregulating miR-33a-5p inhibited, while silencing miR-33a-5p increased invasion **(C)** and migration** (D)** abilities in PCa cells. Error bars represent the mean ± S.D. of three independent experiments. *P < 0.05.

To determine the biological role of miR-33a-5p in bone metastasis of PCa, GSEA was performed based on miRNA expression dataset of PCa from TCGA. The results revealed that low level of miR-33a-5p strongly and positively correlated with EMT-associated gene signatures (Figure S2A and B) and the metastatic propensity (Figure S2C). Then, we further exogenously overexpressed miR-33a-5p and endogeneously silenced miR-33a-5p in VCaP that expressed moderate levels of miR-33a-5p, and only exogenously overexpressed miR-33a-5p in C4-2B cells that expressed a relatively low level of miR-33a-5p via virus transduction (Figure S1). We found that overexpression of miR-33a-5p converted the stick-like or long spindle-shaped mesenchymal phenotype of PC-3 cells to a short spindle-shaped or cobblestone-like epithelial profile (Figure 3A). As C4-2B and VCaP cells were predominated with epithelial phenotypes, TGF-β was used in these cells to induce mesenchymal phenotypes (Figure 3A). Subsequently, we overexpressed miR-33a-5p in TGF-β-treated VCaP and C4-2B cells, and found that the mesenchymal phenotypes of VCaP and C4-2B cells induced by TGF-β were reversed by overexpression of miR-33a-5p (Figure 3A), indicating that miR-33a-5p could inhibit TGF-β-induced changes in cell shape. Western blot analysis showed that overexpression of miR-33a-5p elevated expression of the epithelial marker E-cadherin, but reduced the expression of mesenchymal markers vimentin and fibronectin in PCa cells (Figure 3B). Conversely, silencing miR-33a-5p decreased E-cadherin expression, but enhanced expression of vimentin and fibronectin in VCaP cells (Figure 3B). Invasive and migrating abilities of PCa cells were further evaluated via invasion and migration assays. The results showed that silencing miR-33a-5p promoted, while upregulating miR-33a-5p repressed the invasion and migration abilities of PCa cells (Figure 3C and D). These results indicated that miR-33a-5p inhibits EMT, invasion and migration in PCa cells.

### ZEB1 contributes to reduced expression miR-33a-5p in PCa

To determine the underlying mechanism responsible for the reduced expression of miR-33a-5p in PCa tissues, the deletion frequency of MIR33A was first analyzed in the PCa dataset from TCGA. MIR33A deletion was detected in 55 of all 488 PCa samples (11.3%) (Figure S3A). However, there was no significant difference in the expression levels of PCa tissues with and without deletion of MIR33A (Figure S3B), suggesting that deletion is not involved in the decreased expression of miR-33a-5p in PCa tissues. Next, we analyzed the methylation level of the MIR33A promoter in PCa tissues in the PCa dataset from TCGA, and found no obvious differences in methylation levels between PCa tissues and ANT, as well as between PCa/nBM and PCa/BM (Figure S3C and D). These findings indicated that other mechanisms contribute to the decreased expression of miR-33a-5p in PCa tissues.

**Figure 4:**
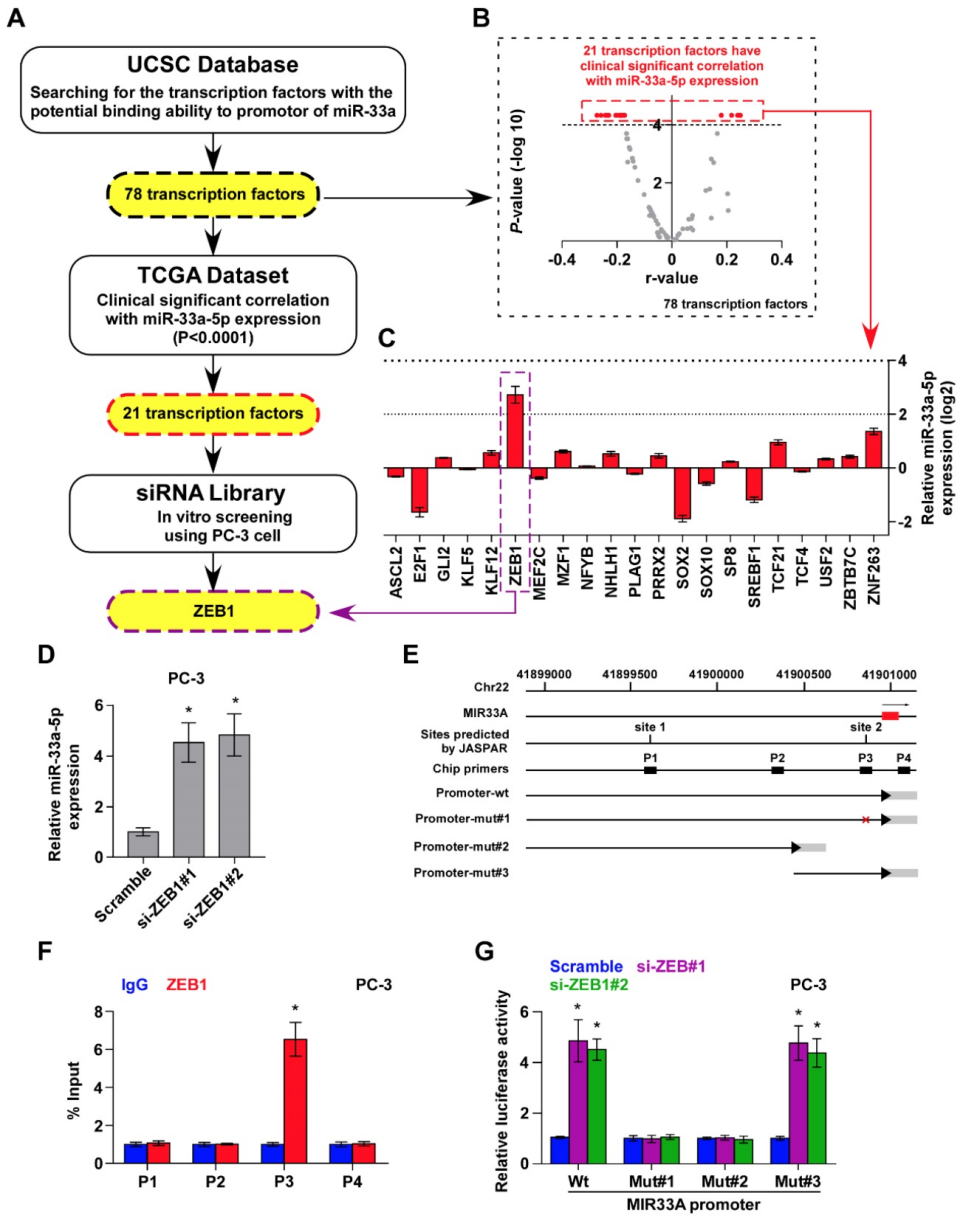
** ZEB1 contributes to miR-33a-5p downexpression in PCa tissues. A,** Hypothetical model illustrates the methods to search for the potential transcriptional factor in regulating miR-33a-5p expression in PCa tissues. **B,** Volcano plot analyzed the clinical correlation of miR-33a-5p expression with 78 transcriptional factors in PCa tissues from TCGA. The red dots represent clinicanlly and significantly correlated transcriptional factors. P < 0.0001. **C,** Identification of the potential transcriptional factor candidate in regulating miR-33a-5p expression by transfecting siRNA of different transcriptional factors. *P < 0.05. **D,** Real-time PCR analysis of miR-33a-5p expression in ZEB1-silenced PC-3 cells. Error bars represent the mean ± S.D. of three independent experiments. *P < 0.05. **E,** Schematic illustration of the promoter regions of MIR33A, the regions containing the primers used for the ChIP assay, as well as the mutant regions of MIR33A promoter. **F,** ChIP analysis of ZEB1 binding sites in the promoter of MIR33A using an anti-ZEB1 antibody or a control IgG respectively in PC-3 cells. *P < 0.05. **G,** The luciferase activity was measured by transfecting wild-type and different types of mutant MIR33A promoter in the indicated PC-3 cells. Error bars represent the mean ± S.D. of three independent experiments. *P < 0.05.

Several lines of evidence have reported that miRNAs are widely regulated at transcriptional level, which is identified as a primary mechanism responsible for aberrant expression of miRNAs in a variety of cancers 24, 46, 47. Therefore, we further analyzed the promoter of MIR33A via UCSC bioinformatics to identify the potential transcription factors with the binding motifs inside the putative promoter region of MIR33A, and found 78 transcription factors with binding motifs within the putative promoter region of MIR33A (Figure 4A). The association of miR-33a-5p expression with these potential transcription factors was further analyzed in PCa dataset from TCGA. The results showed a significant correlation (P < 0.0001) of miR-33a-5p expression with 21 transcription factors (Figure 4A and B). A siRNA library of these 21 transcriptional factors was further used to investigate which ones regulate miR-33a-5p expression. As shown in Figure 4C, we found that miR-33a-5p expression was significantly regulated by multiple transcriptional factors. Among these, ZEB1 was the most important regulator that reduced miR-33a-5p levels with a 6.6-fold change compared with other potential transcription factors (Figure 4C). Consistently, we found that silencing ZEB1 robustly elevated miR-33a-5p expression in three different bone metastatic PCa cell lines (Figure 4D and Figure S4A-F). Surprisingly, expression of the miR-33a-5p host gene, SREBF2, was not affected by downregulating ZEB1 (Figure S4G-I). Indeed, we found multiple ZEB1-binding motifs in the putative promoter region of MIR33A using UCSC (Figure 4E) and JASPAR (Figure S4J). Then, ChIP assay was performed to determine the specific binding regions of ZEB1 within the promoter of MIR33A. As shown in Figure 4F and Figure S4K and L, our results demonstrated that ZEB1 bound to the P3 binding site in the promoter region of MIR33A in PCa cells. Also, enhanced luciferase activity of MIR33A promoter was identified in ZEB1-silencing PCa cells (Figure 4G and Figure S4M and N). However, the luciferase activity of MIR33A promoter was not affected by ZEB1 when the P3 binding site was mutated or truncated (Figure 4G and Figure S4M and N). Therefore, our results demonstrated that ZEB1 transcriptionally inhibits miR-33a-5p expression in PCa tissues.

### ZEB1 upregulation induced by copy number gains inhibits miR-33a-5p in bone metastatic PCa independent of TGF-β signaling

**Figure 5:**
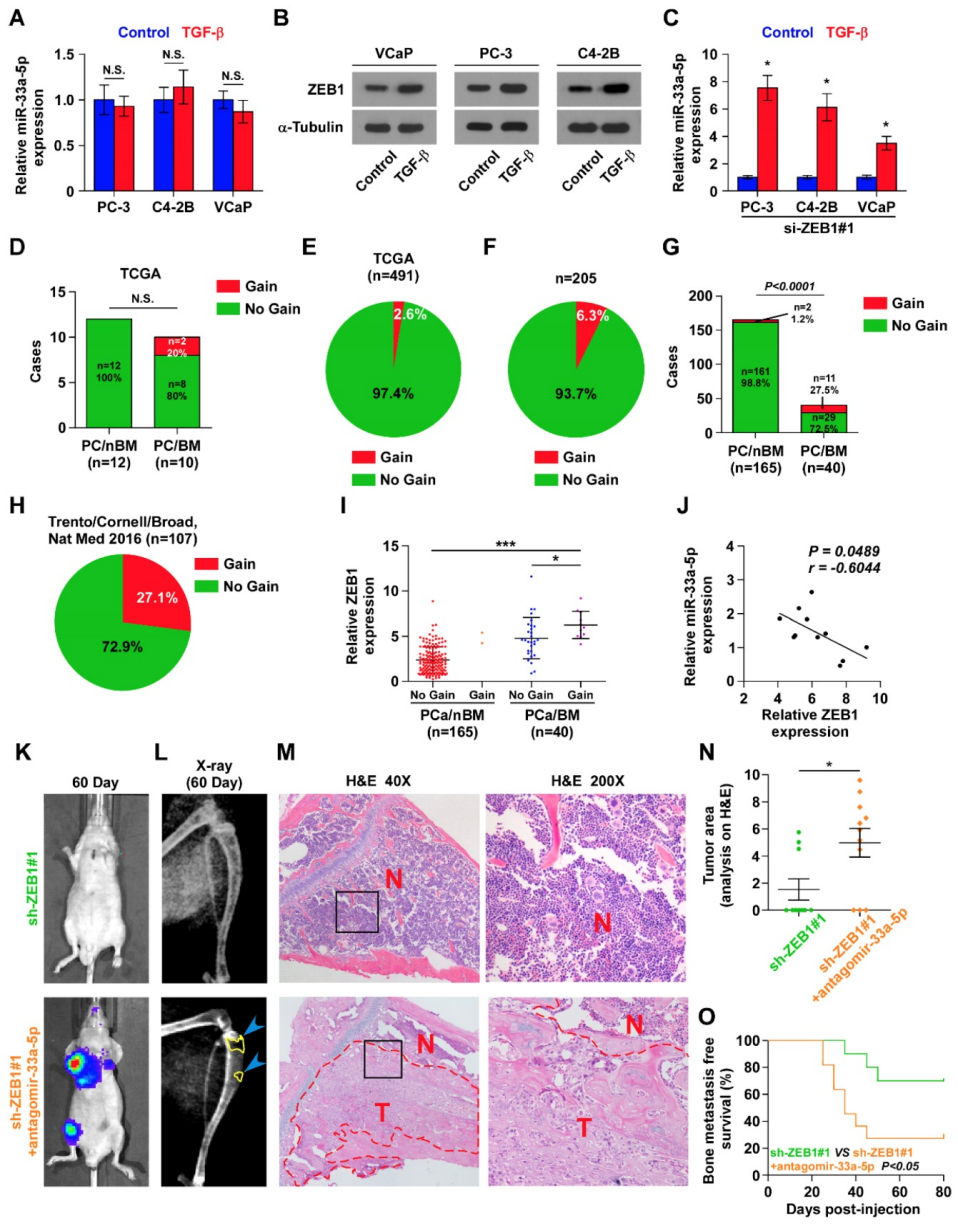
** Gains-induced, not TGF-β signaling-induced ZEB1 upregulation inhibits miR-33a-5p in bone metastatic PCa. A,** Real-time PCR analysis of miR-33a-5p expression in PCa cells treated with TGF-β (5 ng/ml). Transcript levels were normalized by *U6* expression. Error bars represent the mean ± s.d. of three independent experiments. **B,** Western blot analysis of ZEB1 expression in PCa cells treated with TGF-β (5 ng/ml). α-Tubulin served as the loading control.** C,** Real-time PCR analysis of miR-33a-5p expression in ZEB1-silenced PCa cells treated with TGF-β (5 ng/ml). Transcript levels were normalized by *U6* expression. Error bars represent the mean ± s.d. of three independent experiments. *P < 0.05. **D,** Percentages and number of miR-33a-5p samples with gains in PCa patients with different bone metastasis statues in TCGA dataset (n.s. = no significance). **E,** The percentage of miR-33a-5p with gains in the PCa samples from TCGA. **F,** The percentage of miR-33a-5p with gains in our PCa samples. **G,** Percentages and number of miR-33a-5p samples with gains in PCa patients with different bone metastasis statues in our PCa samples. **H,** The percentage of miR-33a-5p with gains in 107 castration resistant neuroendocrine PCa from Trento/Cornell/Broad dataset. **I,** miR-33a-5p expression levels were markedly elevated in PCa/BM with gains compared with that in PCa/BM without gains or PCa/nBM without gains (PCa/nBM without gains, n = 163; PCa/nBM with gains, n = 2; PCa/BM without gains, n = 29; PCa/BM with gains, n = 11). **J,** Correlation of ZEB1 expression with miR-33a-5p expression in 11 separate PCa/BM with gains. **K,** Representative BLIs signal of bone metastasis of a mouse from the indicated groups of mice on day 60.** L,** Representative radiographic images of bone metastases in the mice groups injected with vector and miR-33a-5p-overexpressing PC-3 cells respectively (arrows indicate osteolytic lesions).** M,** Representative H&E-stained sections of tibias from the indicated mouse (T, tumor; N, the adjacent non-tumor tissues). **N,** Histomorphometric analysis of bone osteolytic areas in the tibia of the indicated mice groups. *P < 0.05.** O,** Kaplan-Meier analysis of mouse bone metastasis-free survival in the indicated mice groups.

It is of great important to understand a paradoxical finding that TGF-b treatment did not downregulate miR-33a-5p expression in three individual bone metastatic PCa cell lines (Figure 5A), although expression of ZEB1, a known downstream target gene of TGF-b signaling 48, was induced by TGF-b (Figure 5B). This controversial finding prompted us to posit that other factors regulated by or associated with TGF-b signaling may promote overexpression of miR-33a-5p, giving rise to the net results of miR-33a-5p expression showing no significant change under treatment of TGF-b in PCa cells compared with those without TGF-b treatment. This hypothesis was further supported by our result that TGF-b treatment induced miR-33a-5p overexpression in ZEB1-silenced PCa cells (Figure 5C). Therefore, these results supported the notion that ZEB1 upregulation inhibits miR-33a-5p expression independent on TGF-b signaling.

To explore the underlying mechanism of ZEB1 upregulation-mediated miR-33a-5p downregulation in bone metastatic PCa, we further analyzed the PCa dataset from TCGA, and found copy number gains of ZEB1 in 2/10 bone metastatic PCa tissues (20%), but that was not observed in any non-bone metastatic PCa (Figure 5D). Compared with the relatively high occurrence in bone metastatic PCa, copy number gain of ZEB1 only occurred in 13/491 PCa tissues (2.6%) (Figure 5E), suggesting that gain of ZEB1 may be a more frequent event in bone metastasis of PCa. Since the limited samples analyzed in PCa dataset from TCGA, we, therefore, further examined the gain levels of ZEB1 in a more expanded panel of bone metastatic PCa tissues. Consistently, gains in ZEB1 copy number were found in 13/205 PCa tissues (approximately 6.3%) (Figure 5F), but was in 11/40 bone metastatic PCa tissues (approximately 27.5%) and in 2 out of 165 non-bone metastatic PCa tissues (approximately 1.2%) (Figure 5G). Notably, the high frequency of ZEB1 copy number gain was identified in castration resistant neuroendocrine PCa in the Beltran study (Figure 5H) 49. Thus, our findings, in combination with other studies, demonstrated that ZEB1 gain occurred with a relatively high predilection and may have a crucial function in highly aggressive PCa tissues, including bone metastatic and neuroendocrine PCa. Indeed, the expression levels of ZEB1 in bone metastatic PCa tissues with copy number gains were significantly upregulated compared with non-bone metastatic PCa tissues and even bone metastatic PCa tissues without copy number gains in ZEB1 (Figure 5I). Importantly, a negative correlation of ZEB1 expression with miR-33a-5p expression was observed in bone metastatic PCa tissues with gains (Figure 5J). Thus, these findings supported the idea that ZEB1 upregulation induced by copy number gain plays an important role in bone metastasis of PCa by inhibiting miR-33a-5p. This was particularly true in animal experiment that anti-bone metastatic role of silencing ZEB1 in PCa cells was significantly reversed by injection of antagomir-33a-5p, as indicated by an increased bone metastatic sites, osteolytic area and bone burden, and reduced bone metastasis free survival (Figure 5K-O and Figure S5A-C). Collectively, our results demonstrated that ZEB1 upregulation induced by copy number gain represses miR-33a-5p expression, leading to the development of PCa bone metastasis.

### miR-33a-5p forms a double negative feedback loop with ZEB1 in target-independent manner

**Figure 6:**
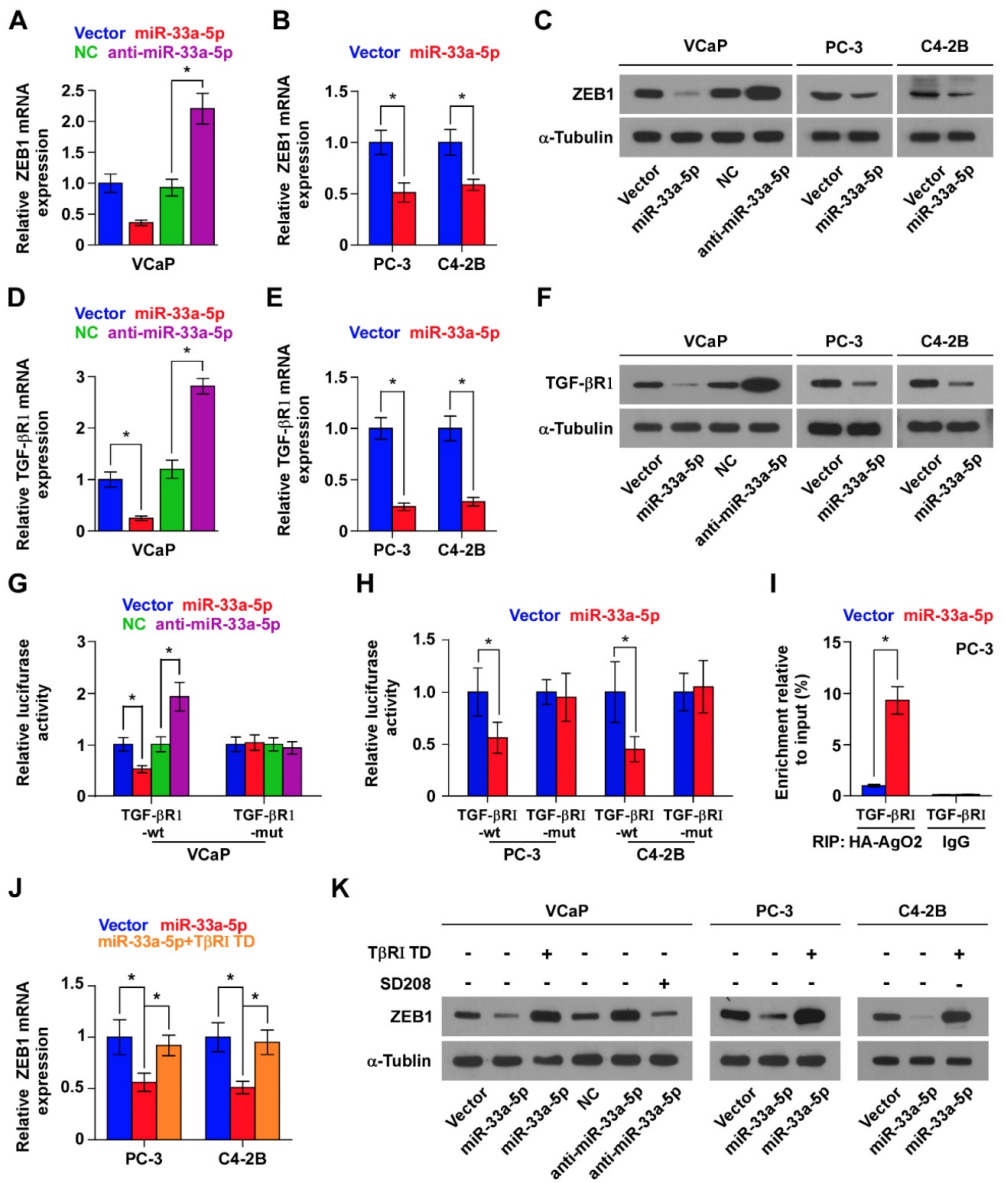
** miR-33a-5p inhibits ZEB1 dependent on TGF-β signaling activity. A and B** Real-time PCR analysis of ZEB1 expression in the indicated cells. Error bars represent the mean ± S.D. of three independent experiments. *P < 0.05.** C,** Western blotting of ZEB1 expression in the indicated cells. α-Tubulin served as the loading control. **D and E,** Real-time PCR analysis of TGFBRI expression in the indicated cells. Error bars represent the mean ± S.D. of three independent experiments. *P < 0.05.** F,** Western blotting of TGFBRI expression in the indicated cells. α-Tubulin served as the loading control. **G and H,** Luciferase assay of cells transfected with pmirGLO-3'UTR reporter of TGFBRI in the miR-33a-5p overexpressing and silencing PCa cells. *P < 0.05.** I,** MiRNP IP assay showing the association between miR-33a-5p and TGFBRI transcripts in PC-3 cells cells. Pulldown of IgG antibody served as the negative control. **J and K,** Real-time PCR and Western blot analysis revealed that TβR1 TD reversed the repressive effect of miR-33a-5p overexpression on ZEB1 expression in PCa cells; SD208 attenuated the stimulatory effects of miR-33a-5p downexpression on ZEB1 expression in VCaP cells. α-Tubulin served as the loading control. *P < 0.05.

Interestingly, we found that ZEB1 may be a potential target of miR-33a-5p (Figure S6A), suggesting the existence of ZEB1-induced the miR-33a-5p-mediating reciprocal repressive feedback loop in bone metastasis of PCa. Indeed, the double-negative feedback loops between ZEB1 and miRNAs have been extensively reported 26, 50. As expected, RT-PCR and Western blot analysis showed that overexpression of miR-33a-5p reduced, while silencing miR-33a-5p increased expression of ZEB1 in PCa cells (Figure 6A-C). Surprisingly, neither the luciferase activity of 3'UTR of ZEB1, nor a direct association of miR-33a-5p with ZEB1 transcript as assessed by microribonucleoprotein (miRNP) immunoprecipitation (IP) assay was affected by overexpressing or downexpressing miR-33a-5p in PCa cells (Figure S6B-G). Collectively, although our findings demonstrated the existence of double negative feedback loop between miR-33a-5p and ZEB1 in PCa cells, ZEB1 is not a *bona fide* target of miR-33a-5p.

### miR-33a-5p inhibits ZEB1 dependent on TGF-β signaling

TGF-β receptor 1 (TGFBRI) that transducts canonical TGF-β signaling after bind to TGF-β ligands 51, 52 is found to be a potential target of miR-33a-5p by analyzing multiple publicly available algorithms, including TargetScan, miRanda and miRWalk (Figure S7A). It has been demonstrated that ZEB1 is a well-documented downstream target gene of TGF-β signaling 48. Hence, we speculated that miR-33a-5p inhibits ZEB1 expression by repressing TGF-β signaling via directly targeting TGFBRI. Undoubtedly, overexpression of miR-33a-5p reduced the expression of TGFBRI (Figure 6D-F), as well as repressed activity of TGF-β signaling, including the decreased transcriptional activity of the TGF-β/Smad-responsive luciferase reporter plasmid CAGA12 and the decreased pSMAD2/3 nuclear translocation (Figure S7B and C). Elevated levels of miR-33a-5p also reduced the expression of multiple downstream bone metastasis-related genes of TGF-β pathway in the absence or presence of ectopic TGF-β in PCa cells (Figure S7D). Conversely, silencing miR-33a-5p increased TGFBRI expression (Figure 6D-F). Furthermore, luciferase assay showed that upregulating miR-33a-5p inhibited, while silencing miR-33a-5p enhanced the reporter activity of 3'UTR of TGFBRI, but not of the mutant 3'UTR of TGFBRI (Figure 6G and H). RIP assay revealed a direct association of miR-33a-5p with TGFBRI transcripts (Figure 6I and Figure S8A and B), supporting the direct repressive effects of miR-33a-5p on TGFBRI. Thus, these results revealed that miR-33a-5p inhibits ZEB1 expression by targeting TGFBRI and repressing TGF-β signaling.

The functional role of TGF-β signaling in miR-33a-5p-induced ZEB1 decreased expression in PCa cells was further investigated. TβR1 TD, a constitutively active mutant TGFBRI plasmid containing a substitution of threonine 204 with aspartic acid (T204D) that results in constitutive activation of TGF-β signaling in the absence of TGF-β stimulation 53, 54, was transfected into miR-33a-5p-overexpressing PCa cells.

Expression of TβR1 TD enhanced activity of TGF-β signaling in miR-33a-5p-overexpressing PCa cells (Figure S8C), as well as reversed the ZEB1 expression repressed by miR-33a-5p overexpression (Figure 6J and K, and Figure S8D). Furthermore, SD208, an inhibitor of the kinase activity of TGFBRI, attenuated the stimulatory effects caused by the decreased expression of miR-33a-5p on TGF-β signaling (Figure S8E) and ZEB1 expression (Figure 6K, and Figure S8D) in VCaP cells. Taken together, these findings demonstrated that miR-33a-5p inhibits ZEB1 expression by repressing TGF-β signaling via directly targeting TGFBRI.

### Reconstitution of TGFBRI reverses bone metastasis of PCa cells repressed by miR-33a-5p overexpression

**Figure 7:**
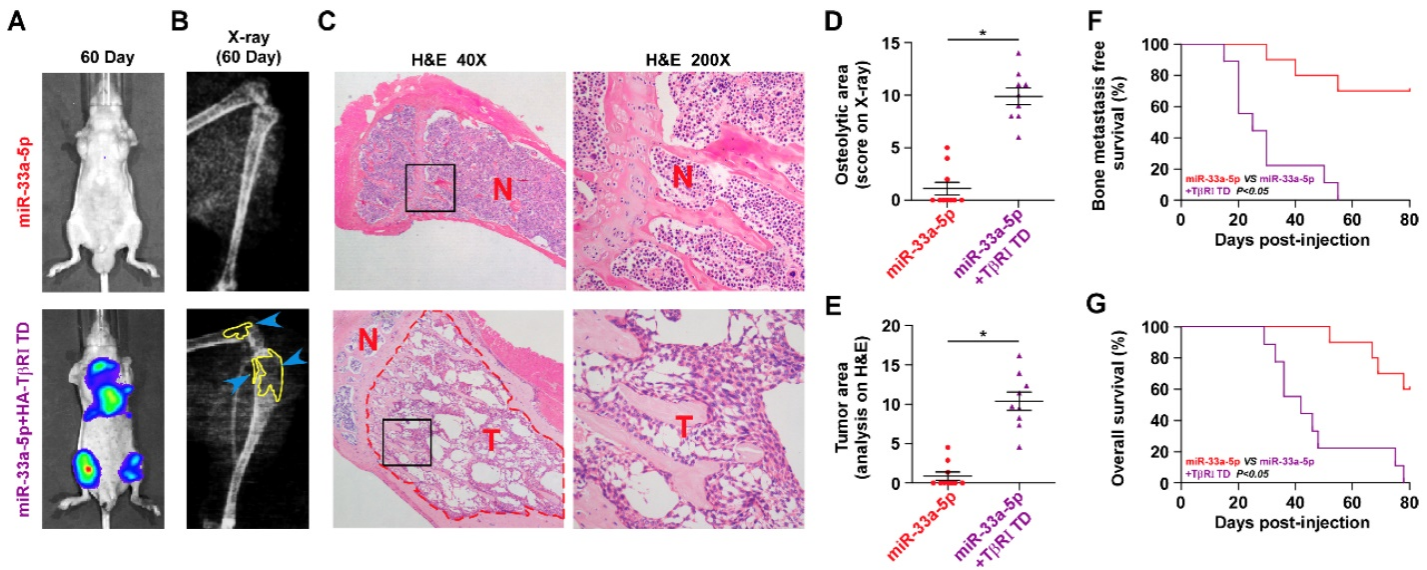
** Reconstitution of TGFBRI increases bone metastasis ability in miR-33a-5p-overexpressing PCa cells. A,** Representative BLI signal of bone metastasis in a mouse from the indicated groups of mice on day 60. **B,** Representative radiographic images of bone metastases in the indicated mice (arrows indicate osteolytic lesions). **C,** Representative H&E-stained sections of tibias from the indicated mouse (T, tumor; N, the adjacent non-tumor tissues). **D,** The sum of bone metastasis scores for each mouse among tumor-bearing mice inoculated cells of miR-33a-5p-overexpression (n = 10) and miR-33a-5p-overexpression plus TGFBRI reconstitution (n = 9). **E,** Histomorphometric analysis of bone osteolytic areas in the tibia of the indicated mice groups. *P < 0.05.** F and G,** Kaplan-Meier analysis of mouse bone metastasis-free **(F)** and overall **(G)** survival in the indicated mice groups.

We further determined the functional significance of TGF-β signaling in the anti-bone metastatic role of miR-33a-5p in PCa cells *in vitro* and* in vivo*. As shown in Figure S9A and B, TβR1 TD reversed the inhibitory effects of miR-33a-5p overexpression on invasion and migration of PCa cells *in vitro*. More importantly, TβR1 TD markedly attenuated the anti-bone metastatic role of miR-33a-5p *in vivo*, as demonstrated by the increased bone metastatic sites, osteolytic area and bone burden, and reduced bone metastasis-free survival (Figure 7A-G). Collectively, these findings indicated that restoration of TGFBRI is sufficient to reverse the inhibitory effects of miR-33a-5p overexpression on bone metastasis.

### Clinical correlation of miR-33a-5p with TGFBRI, TGF-β signaling activity and ZEB1 in human PCa tissues

**Figure 8:**
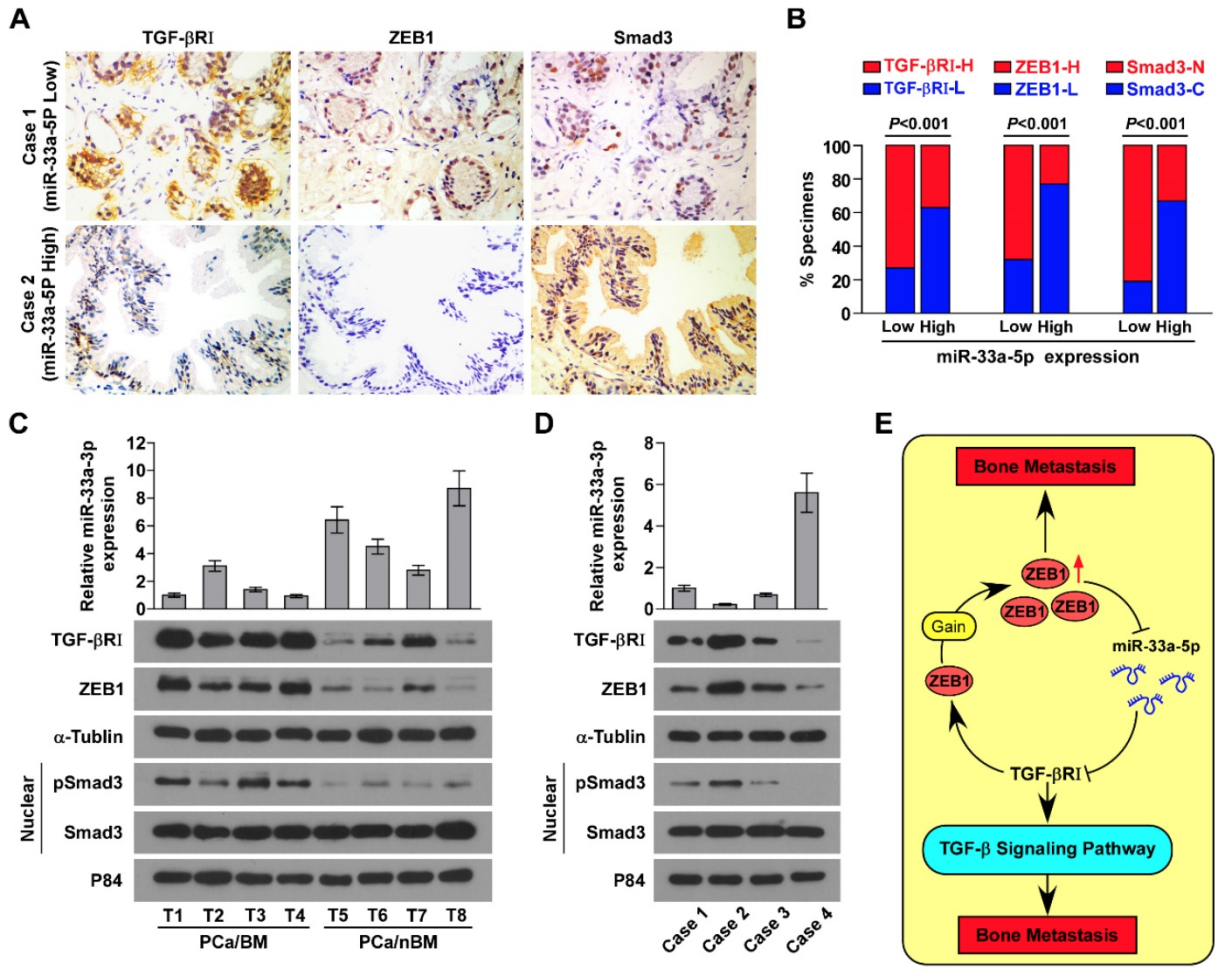
** Clinical relevance between miR-33a-5p and TGFBRI, ZEB1 and TGF-β signaling activity in PCa. A,** miR-33a-5p expression levels were inversely associated with expression of TGFBRI, ZEB1 and nuclear SMAD3 expression in 205 primary PCa tissues. Two representative cases (Low and High miR-33a-5p). Scale bars, 50 μm. **B,** Percentage of specimens showing low- or high expression of TGFBRI and ZEB1 intensity, and nuclear or cytoplasmic SMAD3 in patient specimens, respectively, with low and high miR-33a-5p expression.** C,** Real-time PCR and western blot analysis of miR-33a-5p and TGFBRI, ZEB1 and pSMAD3 expression in 4 random PCa/BM with gains (T1-T4) and 4 random PCa/nBM without gains (T5-T8). U6 was used as the control for miR-33a-5p loading. miR-33a-5p expression levels were normalized to that miR-33a-5p expression of T1. Each bar represents the mean ± SD of three independent experiments. Loading controls were α-tubulin and p84 for the cytoplasmic and nuclear fractions. **D,** Real-time PCR and western blot analysis of miR-33a-5p and TGFBRI, ZEB1 and pSMAD3 expression in 4 individual primary PCa cells. U6 was used as the control for miR-33a-5p loading. miR-33a-5p expression levels were normalized to that miR-33a-5p expression of sample 1. Each bar represents the mean ± SD of three independent experiments. Loading controls were α-tubulin and p84 for the cytoplasmic and nuclear fractions. **E,** Hypothetical model illustrating that formation of reciprocal repressive feedback loop between miR-33a-5p and ZEB1 depend on activity of TGF-β signaling in bone metastasis of PCa.

The clinical relevance of miR-33a-5p expression with TGFBRI, TGF-β signaling activity and ZEB1 was investigated in clinical PCa tissues. Immunohistochemistry (IHC) analysis showed that miR-33a-5p expression inversely correlated with TGFBRI, ZEB1 and nuclear SMAD3 expression in clinical PCa specimens (Figure 8A and B). Furthermore, miR-33a-5p expression and TGFBRI, pSMAD3 and ZEB1 protein expression were further examined by RT-PCR and western blot analysis, respectively, in 4 random bone metastatic PCa tissues with gains (T1-T4) and 4 random non-bone metastatic PCa tissues without gains (T5-T8). As shown in Figure 8C, expression of TGFBRI, pSMAD3 and ZEB1 were upregulated in PCa/BM (T1-4) compared with those in PCa/nBM (T5-T8). Conversely, miR-33a-5p expression displayed an opposite pattern (Figure 8C). Pearson analysis revealed that miR-33a-5p expression inversely correlated with TGFBRI, pSMAD3 and ZEB1 (Figure S10A-C). Importantly, the negative correlation of miR-33a-5p expression with TGFBRI, ZEB1 and pSMAD3 was also demonstrated in our primary PCa cells that were isolated and cultured in our previous study (Figure 8D) 44. Taken together, these results demonstrated that ZEB1 upregulation induced by the copy number gain mediates the reciprocal repressive feedback loop with miR-33a-5p, which further regulates bone metastasis of PCa dependent on TGF-β signaling (Figure 8E).

## Discussion

The primary findings of this current study provide critical insights into the crucial role of miR-33a-5p/ZEB1 negative feedback loop in EMT and bone metastasis of PCa. We have shown that copy number gain of ZEB1 contributed to decreased expression of miR-33a-5p, which further promoted EMT, invasion, migration and bone metastasis of PCa cells. In turn, ZEB1 expression was inhibited by miR-33a-5p. Our results further demonstrated that miR-33a-5p did not directly target ZEB1 expression, but reduced ZEB1 expression by inhibiting TGF-β signaling via targeting TGFBRI, indicating that formation of an indirect double-negative feedback loop between miR-33a-5p and ZEB1 was dependent on TGF-β signaling. Thus, our results uncover a novel molecular mechanism of the formation of the double-negative feedback loop between miR-33a-5p and ZEB1 involving TGF-β signaling.

miR-33a-5p has been reported to be downregulated in several human cancers, and its low expression significantly correlated with progression, metastasis and poor survival 27-31. However, miR-33a-5p has also been shown to be upregulated in glioblastoma and multiple myeloma 55, 56. These findings indicated that miR-33a-5p plays an opposite, or even paradoxical role depending on cancer types. In PCa, miR-33a-5p was found to be downregulated in PCa tissues, and ectopic expression of miR-33a-5p repressed proliferation and invasion of PCa cells in two independent studies 32, 33, supporting the notion that miR-33a-5p plays a tumor-suppressive role in PCa. We previously reported that miR-33a-5p was significantly downregulated in bone metastatic tissues in the miRNA microarray dataset 45, implicating low expression of miR-33a-5p in bone metastasis of PCa. However, the clinical significance and functional role of miR-33a-5p in bone metastasis of PCa have not yet been elucidated. In the present study, our results showed that miR-33a-5p was dramatically downregulated in PCa tissues with bone metastasis, which positively correlated with poor clinicopathological features and predicted poor bone metastasis-free survival in PCa patients. Upregulating miR-33a-5p inhibited, while silencing miR-33a-5p enhanced EMT, invasion and migration of PCa cells. More importantly, overexpression of miR-33a-5p repressed bone metastasis of PCa cells *in vivo*. Collectively, our findings identified the anti-bone metastatic role of miR-33a-5p in PCa.

As mentioned above, decreased expression of miR-33a-5p has been extensively reported in the vast majority of cancers. However, the underlying mechanism responsible for the downregulation of miR-33a-5p in cancers remains to be clarified. In this study, the analysis of the TCGA data showed that neither deletion nor methylation contributed to the reduced expression of miR-33a-5p in PCa. Our results further revealed that ZEB1-mediated transcriptional repression resulted in the downregulation of miR-33a-5p in bone metastatic PCa. Indeed, upregulation of ZEB1 has been extensively reported to be pivotal in the metastasis of cancer cells 12, 13. However, the molecular details for ZEB1 upregulation need to be further elucidated. Although ZEB1 is a critical downstream target gene of TGF-β signaling 48, our results showed its upregulation was not due to TGF-β signaling but was a consequence of the gain in its copy number. To the best of our knowledge, ZEB1 has rarely been reported to be amplified in the context of cancer. Notably, Beltran et al have found a high frequency of ZEB1 gain (27.1%) in castration resistant neuroendocrine PCa 49, indicating that copy number gain of ZEB1 may be more commonly observed in malignant progression of PCa. In this study, we detected that ZEB1 gains were more prevalent in bone metastatic PCa compared with that in non- bone metastatic PCa and all types of PCa, suggesting the copy number gain of ZEB1 to be a characteristic feature of metastatic PCa. More significantly, silencing ZEB1 induced miR-33a-5p overexpression and repressed the development of bone metastasis of PCa, which was reversed by antagomir-33a-5p. Thus, our results demonstrate that copy number ZEB1 upregulation induced by copy number gain represses miR-33a-5p expression, leading to the development of PCa bone metastasis, as well as suggest that miR-33a-5p hold a promising therapeutic efficacy against ZEB1-induced bone metastasis of PCa.

Indeed, miRNAs-based therapies in metastatic cancer represent an area of intense investigation. A study from Cai and colleagues has demonstrated the therapeutic effect of miR-128-3p inhibition in chemoresistance-associated NSCLC metastasis by both miRNA sponge and antagomir strategies *in vivo* 57. Notably, Tang et al have reported that tail vein injection of agomir-133a-3p significantly repressed bone metastasis of PCa *in vivo* 58. Therefore, miRNAs-centered therapeutic strategies could serve as a promising alternative for surgical methods, particularly for metastatic cancer, where the options of surgical treatment are limited so far. Here, we reported that overexpression of miR-33a-5p inhibited bone metastasis of PCa, and antagomir-33a-5p significantly reversed the inhibitory effects of ZEB1 downregulation on bone metastasis of PCa *in vivo*. Therefore, our findings support the notion that miR-33a-5p may be used as a potential anti-bone metastatic therapeutic approach for PCa, particularly in ZEB1-induced bone metastasis of PCa.

Previously, a reciprocal repressive feedback loop has been reported between ZEB1 and miRNAs. A study from Burk et al has shown that a reciprocal negative loop between miR-141 and miR-200c and ZEB1 promotes EMT and invasion in multiple cancers 26. Furthermore, Chen and colleagues have found that miR-200c/ZEB1 reciprocal axis-mediated CD8^+^ T cell immunosuppression promoted metastasis of lung cancer 14. In the present study, we have shown ZEB1 to be a target of miR-33a-5p and provided evidence for the existence of a reciprocal repressive loop between ZEB1 and miR-33a-5p. More significantly, our data further revealed that miR-33a-5p inhibited ZEB1 expression in a target-independent manner by repressing TGF-β signaling via directly targeting TGFBRI. These data support the notion that reciprocal repression between miR-33a-5p and ZEB1 in PCa depends on TGF-β signaling.

Besides the well-documented direct double negative feedback loop between ZEB1 and miR-200 family members 14, 26, to the best of our knowledge, our current study, for the first time, provided evidence of an indirect and reciprocal repressive loop between ZEB1 and miR-33a-5p in prostate cancer (PCa) bone metastasis, where we have shown that the copy number gain of ZEB1 transcriptionally inhibited miR-33a-5p expression; in turn, miR-33a-5p inhibited ZEB1 expression in a target-independent manner but was dependent on TGF-β signaling, which mediated an indirect and reciprocal repressive loop between ZEB1 and miR-33a-5p in PCa. Collectively, our results clarify the complex molecular underpinning of TGF signaling pertaining to the miR-33a-5p/ZEB-1 negative feedback loop.

Another interesting finding presented in our study was that TGF-β treatment did not have an influence on miR-33a-5p expression in PCa cells, although ZEB1 expression was remarkably induced by TGF-β. As a TGF-β signaling-dependent downstream transcriptional factors, ZEB1 has been demonstrated to transcritionally inhibit miR-33a-5p expression in PCa cells. This finding implied that some other factors regulated by or associated with TGF-β signaling may be implicated in positive regulation of miR-33a-5p, leading to unchanged expression of miR-33a-5p under treatment of TGF-β. Our results further demonstrated that TGF-β treatment upregulated miR-33a-5p overexpression in ZEB1-silenced PCa cells. In this scenario, which factor or factors regulated by or associated with TGF-β signaling is implicated in this TGF-β-induced miR-33a-5p overexpression, which needs to be further investigated in our following work.

## Conclusions

In summary, our results demonstrated that transcriptional repression of miR-33a-5p by copy number gain in ZEB1 promoted bone metastasis of PCa by activating TGF-β signaling. Our data further elucidated the important role of double-negative feedback loop between miR-33a-5p and ZEB1 in bone metastasis of PCa. Therefore, our study reinforce our understanding that miR-33a-5p forms a regulatory motif with ZEB1 through a more complex negative feedback loop, which underlies the pathogenesis of PCa bone metastasis.

## Supplementary Material

Supplementary figures and tables.
