# Supplementary material for: Copy number gain of ZEB1 mediates a double-negative feedback loop with miR-33a-5p that regulates EMT and bone metastasis of prostate cancer dependent on TGF-β signaling
Source: Theranostics. 2019 Aug 14;9(21):6063–79. doi: 10.7150/thno.36735 (PMC6735523; doi:10.7150/thno.36735)
Supplement: Supplementary file 1 — Supplementary figures and tables. [file thnov09p6063s1.pdf]

Supplementary Information

Table S1. A list of primers used in the reactions for clone PCR.

**Primer**	
miR-33a-5p-clone-F	CATTTGCTCCAGCGGTTTGC
miR-33a-5p-clone-R	GGGAGTGGCTGAGAATCTGAATC
ALK5-CDS-F	GCCACCATGGAGGCGGCGGTC
ALK5-CDS-R	TTACATTTTGATGCCTTCCTGTTGAC
ALK5-T204D-F	TTGCGAGAGATATTGTGTTACAAGAAAGCATTGGC
ALK5-T204D-R	ACACAATATCTCTCGCAATTGTTCTCTGAACAAGC
ZEB1-3'UTR-F	TTGTACTGTATGTCTTCAAACCTGGC
ZEB1-3'UTR -R	AGGCAGTGAGGAATGTGAGAG
TGFBR1-3'UTR-F	GTAAACGTTCGGTGGATCCTCTG
TGFBR1-3'UTR -R	GGATGGACCAGGGATGTCTATGC

Table S2. List of primers used for real-time RT-PCR.

**Primer**	
ZEB1-up	GGCATACACCTACTCAACTACGG
ZEB1-dn	TGGGCGGTGTAGAATCAGAGTC
SREBF2-up	CTCCATTGACTCTGAGCCAGGA
SREBF2-dn	GAATCCGTGAGCGGTCTACCAT
TGF-βR1-up	GACAACGTCAGGTTCTGGCTCA
TGF-βR1-dn	CCGCCACTTTCCTCTCCAAACT
IL11-up	TGAAGACTCGGCTGTGACC
IL11-dn	CCTCACGGAAGGACTGTCTC
PTHRP-up	ACTCGCTCTGCCTGGTTAGA
PTHRP-dn	GGAGGTGTCAGACAGGTGGT
CTGF-up	GCTACCACATTTCCTACCTAGAAATCA
CTGF-dn	GACAGTCCGTCAAAACAGATTGTT
NEDD9 -up	CTACAGGGTAAGGAGGAGTTT
NEDD9-dn	TGGGTCTCACATTGGTCAT
MMP13-up	AACATCCAAAAACGCCAGAC
MMP13-dn	GGAAGTTCTGGCCAAAATGA
ADAM19-up	TTTCTCAGAACAGCGGGACT
ADAM19-dn	TGTTGATCACCTTTCGCTTG
THBS1-up	TTGTCTTTGGAACCACACCA
THBS1-dn	TTGTCAAGGGTGAGGAGGAC
COL1A1-up	CCTGGATGCCATCAAAGTCT
COL1A1-dn	CGCCATACTCGAACTGGAAT
VEGFA-up	AAGGAGGAGGGCAGAATCAT
VEGFA-dn	CACACAGGATGGCTTGAAGA
GAPDH-up	ATTCCACCCATGGCAAATTC
GAPDH-dn	TGGGATTTCCATTGATGACAAG

Table S3. Clinicopathological features of 205 prostate cancer patients

Parameters	Number of cases
Age (years)	
<72	102
⩾72	103
Median	72
Differentiation	
Well/moderate	90
Poor	115
Serum PSA at diagnosis, μg/ml	
<69.2	102
>69.2	103
Median	69.2
SD	561.1
Mean	224.9
Gleason grade	
⩽7	105
>7	100
miR-33a-5p expression	
<4.512	103
>4.512	102
Median	4.5119
SD	2.065
Mean	4.236
ZEB1 expression	
<2.4823	102
>2.4823	103
Median	2.4823
SD	1.971
Mean	2.945
BM status	
nBM	165
BM	40

Abbreviation: PSA, Prostate-specific Antigen; SD, Standard Deviation; BM, Bone Metastasis; n-BM: Non-bone Metastasis.

Table S4. List of primers used for ChIP assay

**Primer**	
P1-F	AGCACTCTGACTAGGTAAGC
P1-R	TCTGGAGCGAGGTGACTTTG
P2-F	CATGGAAGATGGCCTTCACC
P2-R	GTGGCAGCATTGGAGTCAAC
P3-F	TTTCTTAGGCTCCCAGAGTC
P3-R	TGGAGCAAATGCAGGTGAAC
P4-F	CCTGGGATGGCTGTGACTTG
P4-R	ACTGAAGGCCCTATCAGGTG

Table S5. Relationship between miR-33a-5p and clinicopathological features in 205 patients with prostate cancer.

Parameters	Number of cases	miR-33a-5p expression		P-values
		Low	High	
Age (years)				
<72	102	59	47	
⩾72	103	44	55	0.125
Differentiation				
Well/moderate	90	23	67	
Poor	115	80	35	<0.001*
Serum PSA				
<69.2	102	34	68	<0.001*
⩾69.2	103	69	34	
Gleason grade				
⩽7	105	29	76	
>7	100	74	26	<0.001*
BM status				
nBM	165	66	99	
BM	40	37	3	<0.001*

Table S6. Cox regression univariate and multivariate analyses of prognostic factors for overall survival

Variable	Univariate analysis			Multivariate analysis		
	P-value	Relative risk	95% CI	P-value	Relative risk	95% CI
Age<br>≤71<br>>71	0.818	1.10	0.49-2.48	Not included		
Differentiation<br>Well/moderate<br>Poor	0.016	2.82	1.26-6.30	Not included		
Serum PSA, μg/ml<br>≤63.1<br>>63.1	0.016	2.70	1.21-6.04	/	/	/
Gleason grade<br>≤7<br>>7	0.084	2.06	0.91-4.66	/	/	/
miR-33a-5p expression<br><4.8589<br>≥4.8589	0.014	0.36	0.16-0.81	0.02	0.33	0.13-0.84

"/" means variables not in the Equation

Table S7. Cox regression univariate and multivariate analyses of prognostic factors for bone metastasis-free survival

Variable	Univariate analysis			Multivariate analysis		
	P-value	Relative risk	95% CI	P-value	Relative risk	95% CI
Age<br>≤71<br>>71	0.046	2.04	1.01-4.09	0.002	3.34	1.58-7.04
Differentiation<br>Well/moderate<br>Poor	0.001	3.42	1.71-6.86	Not included		
Serum PSA, μg/ml<br>≤63.1<br>>63.1	0.143	1.68	0.84-3.37	Not included		
Gleason grade<br>≤7<br>>7	0.002	3.11	1.52-6.34	0.019	2.06	1.18-6.01
miR-33a-5p expression<br><4.8589<br>≥4.8589	0.002	0.33	0.16-0.66	0.021	0.35	0.14-0.85

"/" means variables not in the Equation

Supplementary Figures

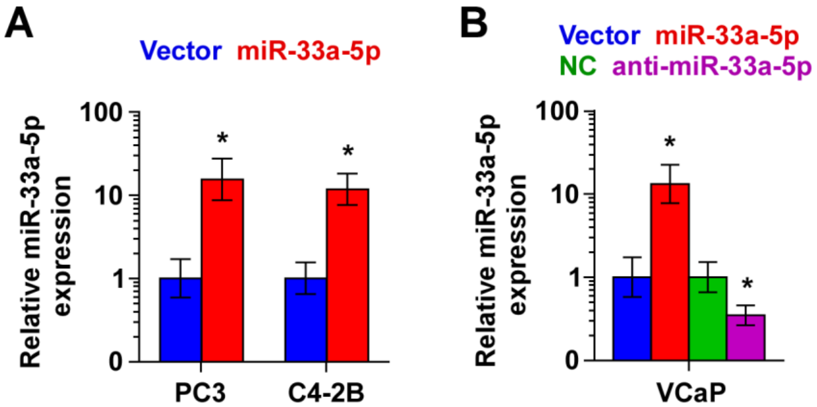

Figure S1. A, Real-time PCR analysis of miR-33a-5p expression in PC-3 and C4-2B cells transduced with miR-33a-5p compared to controls. Transcript levels were normalized by *U6* expression. Error bars represent the mean ± s.d. of three independent experiments. *$P < 0.05$. **B,** Real-time PCR analysis of miR-33a-5p expression inVCaP cells transduced with miR-33a-5p or transfected with anti-miR-33a-5p compared to controls. Transcript levels were normalized by *U6* expression. Error bars represent the mean ± s.d. of three independent experiments. *$P < 0.05$.

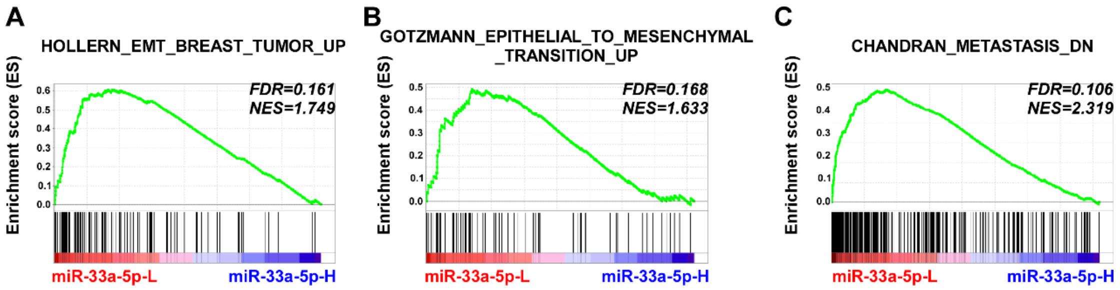

Figure S2. A and B, Gene set enrichment analysis (GSEA) revealed that downexpression of miR-33a-5p significantly and positively correlated with the EMT signatures. **C,** GSEA revealed that downexpression of miR-33a-5p significantly and positively correlated with metastatic propensity.

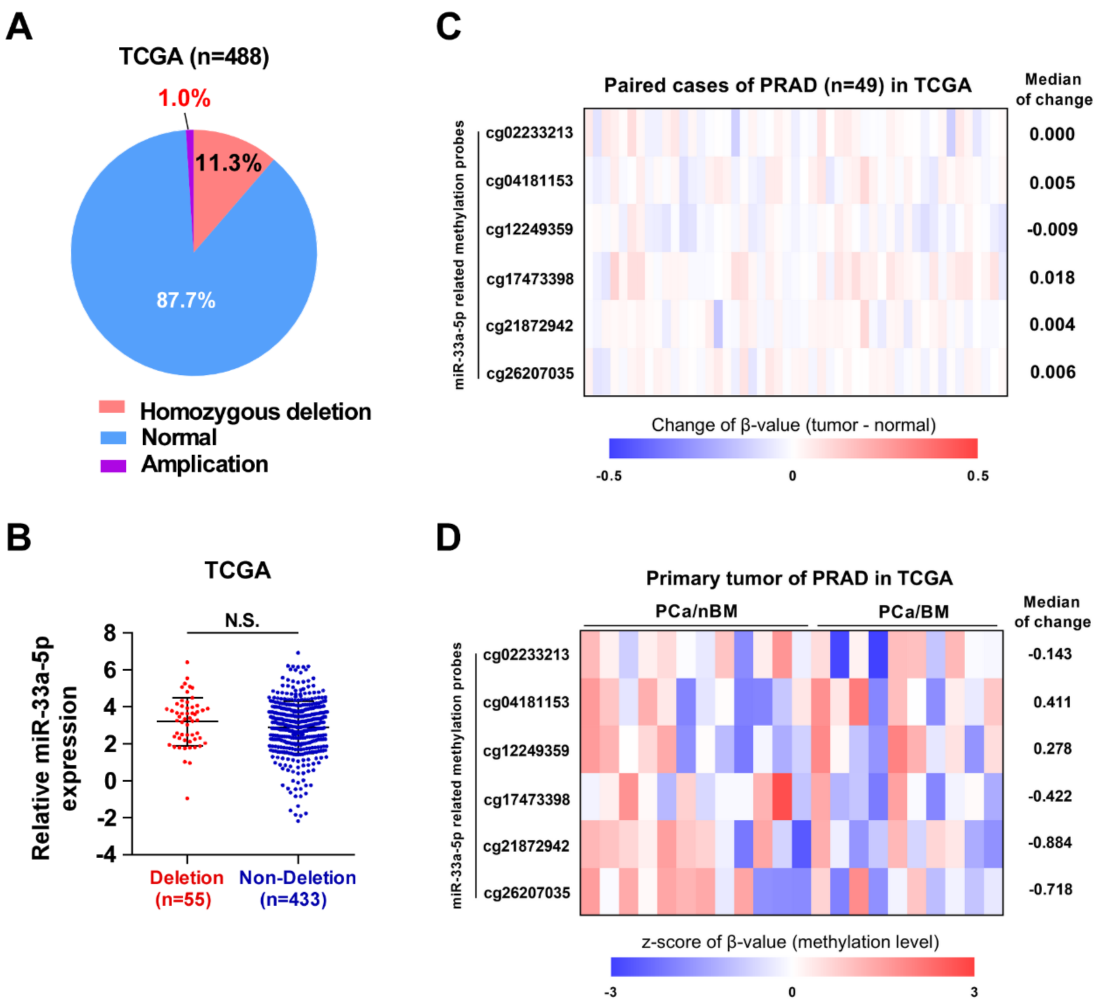

Figure S3. A, Deletion and amplification levels of MIR33A in the PCa dataset from TCGA. **B,** miR-33a-5p expression levels in PCa tissues with deletion and in those without deletion in the PCa dataset from TCGA. **C,** Methylation levels of MIR33A in 49 paired PCa tissues from the TCGA dataset. **D,** Methylation levels of MIR33A between PCa/BM and PCa/nBM tissues from the TCGA dataset.

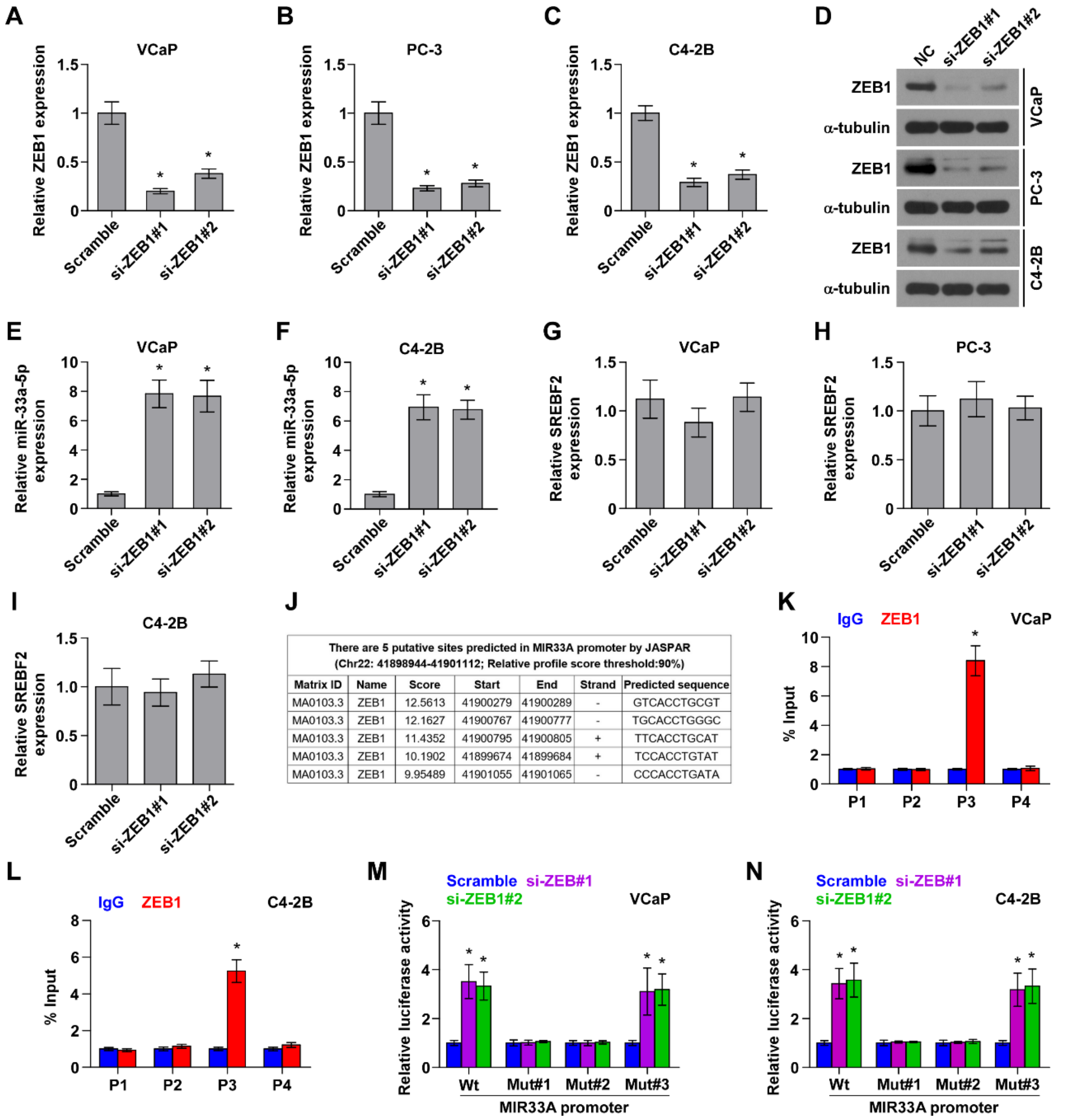

There are 5 putative sites predicted in MIR33A promoter by JASPAR (Chr22: 41898944-41901112; Relative profile score threshold:90%)						
Matrix ID	Name	Score	Start	End	Strand	Predicted sequence
MA0103.3	ZEB1	12.5613	41900279	41900289	-	GTCACCTGCGT
MA0103.3	ZEB1	12.1627	41900767	41900777	-	TGCACCTGGGC
MA0103.3	ZEB1	11.4352	41900795	41900805	+	TTCACCTGCAT
MA0103.3	ZEB1	10.1902	41899674	41899684	+	TCCACCTGTAT
MA0103.3	ZEB1	9.95489	41901055	41901065	-	CCCACCTGATA

Figure S4. A-D, Real-time PCR and Western blot analysis of ZEB1 expression in the indicated PCa cells. α-Tubulin served as the loading control. Error bars represent the mean ± S.D. of three independent experiments. *$P < 0.05$. **E and F,** Real-time PCR analysis of miR-33a-5p expression in the indicated VCaP and C4-2B cells. Error bars represent the mean ± S.D. of three independent experiments. *$P < 0.05$. **G-I,** Real-time PCR analysis of SREBF2 expression in the indicated PCa cells. Error bars represent the mean ± S.D. of three independent experiments. *$P < 0.05$. **J,** The utative binding site of ZEB1 in the promoter of MIR33A by analyzing JASPAR. **K, and I,** ChIP analysis of ZEB1 binding sites in the promoter of MIR33A using an anti-ZEB1 antibody or a control IgG respectively in VCaP **(K)** and C4-2B **(I)** cells. *P < 0.05. **M and N,** The luciferase activity was measured by transfecting wild-type and different types of mutant MIR33A promoter in the indicated VCaP **(M)** and C4-2B **(N)** cells.

Error bars represent the mean ± S.D. of three independent experiments. *$P < 0.05$.

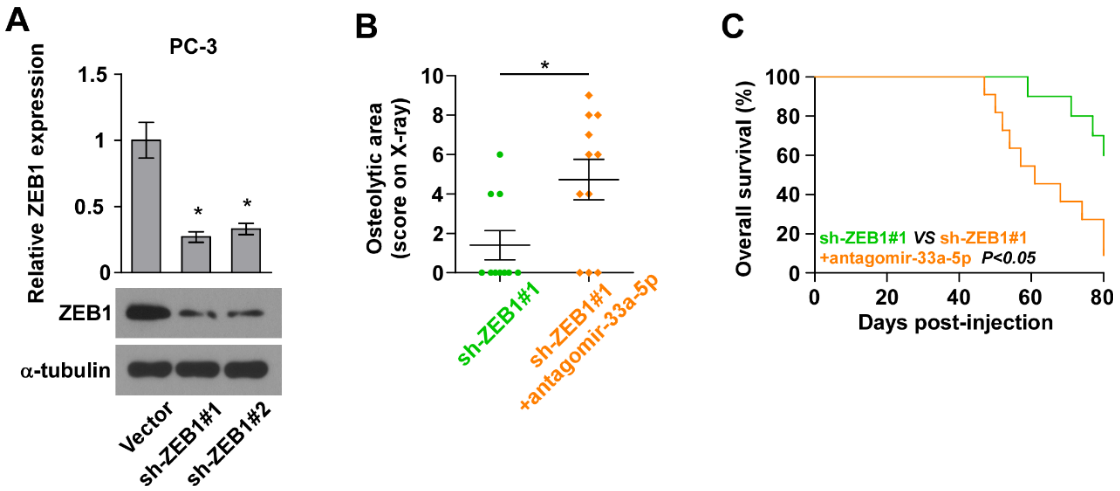

Figure S5. A, Western blot analysis of ZEB1 expression in ZEB1-stably downexpressing PC-3 cells. α-Tubulin served as the loading control. **B,** The sum of bone metastasis scores for each mouse in tumor-bearing mice inoculated with ZEB1-silencing cells (n = 10) or ZEB1-silencing cells plus antagomir-33a-5p (n = 11). **C,** Kaplan-Meier analysis of mouse bone metastasis-free overall survival in the indicated mice groups.

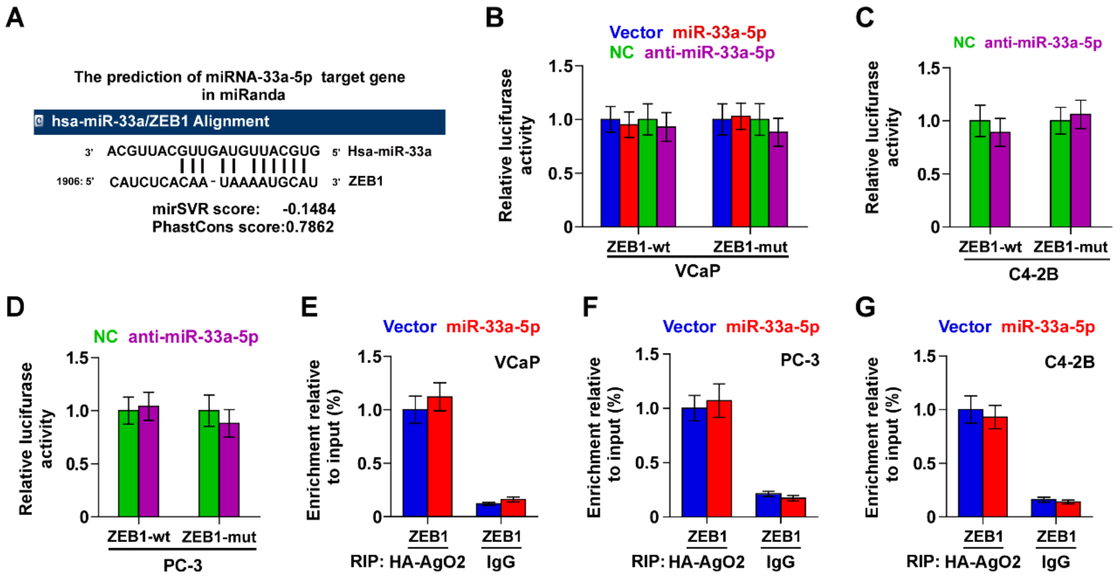

Figure S6. A, Predicted miR-33a-5p targeting wild-type sequences in 3'UTR s of ZEB1. **B-D,** Luciferase assay of cells transfected with pmirGLO-3'UTR reporter of ZEB1 in the miR-33a-5p overexpressing and silencing PCa cells. *$P < 0.05$. **E-G,** MiRNP IP assay showing the association between miR-33a-5p and ZEB1 transcripts in PCa cells cells. Pulldown of IgG antibody served as the negative control.

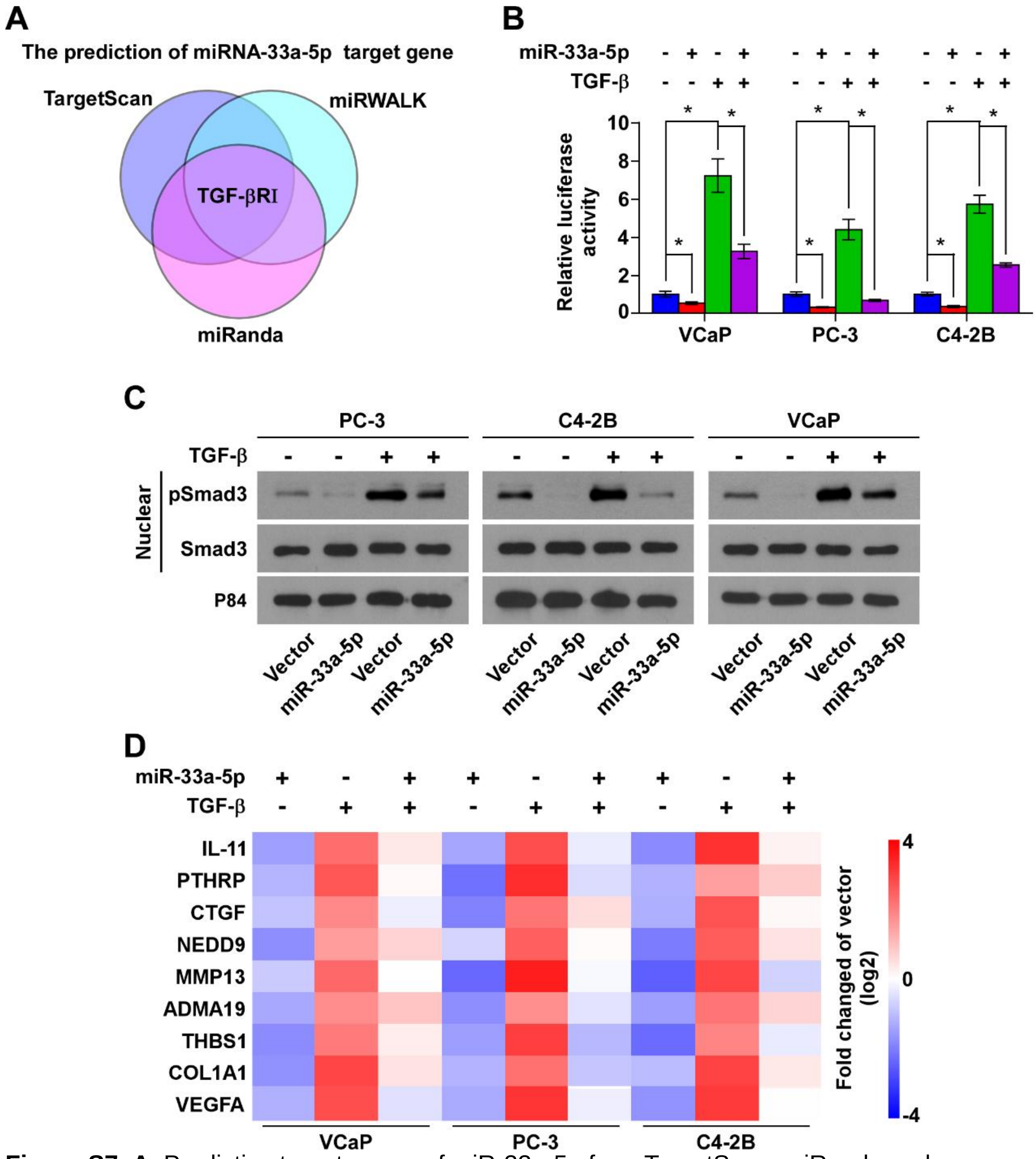

Figure S7. A, Predictive target genes of miR-33a-5p from TargetScan, miRanda and miRWALK. **B,** The effects of miR-33a-5p upregulation on transcriptional activity of TGF-β signaling based on a TGF-β/Smad-responsive luciferase reporter in the absence or presence of TGF-β (5 ng/ml). *P < 0.05. **C,** Western blot analysis showing that upregulation of miR-33a-5p decreased nuclear translocation of pSMAD3 in PCa cells in the absence or presence of TGF-β. The nuclear protein p84 was used as a nuclear protein marker. **D,** Real-time PCR analysis of downstream bone metastasis-related genes of the TGF-β pathway, including CTGF, PTHRP, IL-11, NEDD9, MMP13, ADAM19, THBS1, COL1A1 and VEGFA, in the indicated cells in the absence or presence of TGF-β. Transcript levels were normalized by GAPDH expression.

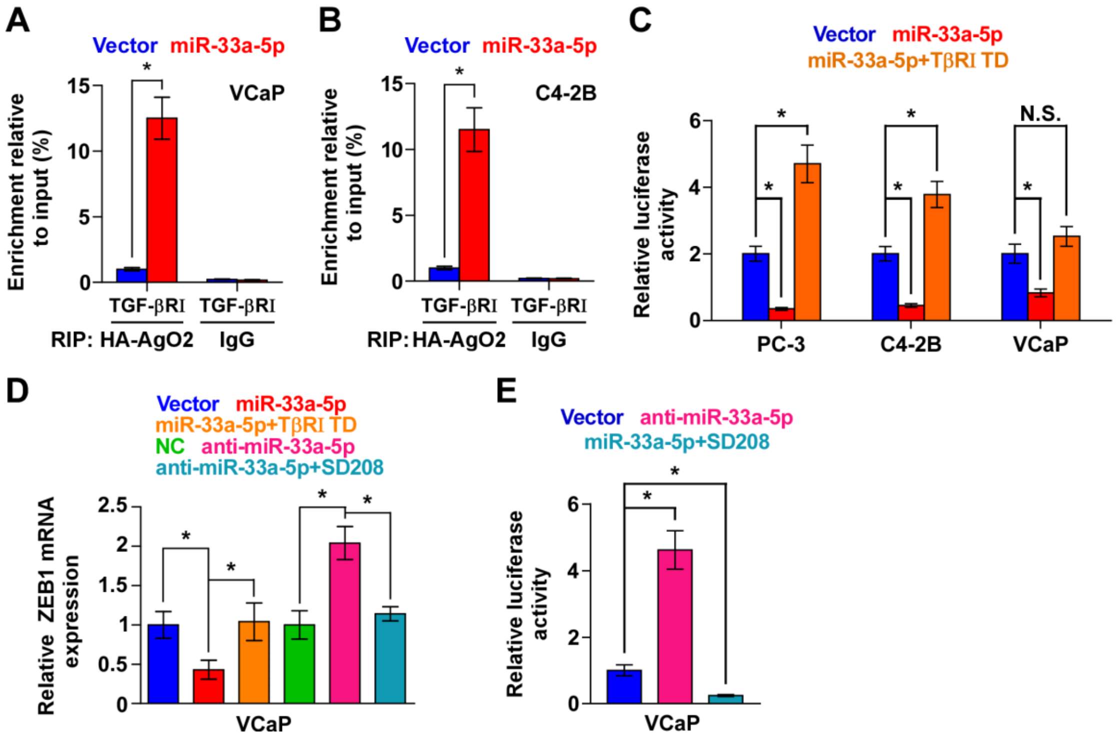

Figure S8. A and B, MiRNP IP assay showing the association between miR-33a-5p and TGFBRI transcripts in VCaP and C4-2B cells. Pulldown of IgG antibody served as the negative control. **C,** The inhibitory effects of miR-33a-5p overexpression on the transcriptional activity of a TGF-β/Smad-responsive luciferase reporter in PCa cells were attenuated by reconstitution of TGFBRI. Error bars represent the mean ± S.D. of three independent experiments. *P < 0.05. **D,** Real-time PCR analysis ZEB1 expression in the indicated VCaP cells. *P < 0.05. **E,** The stimulatory effects of miR-33a-5p downregulation on the transcriptional activity of a TGF-β /Smad-responsive luciferase reporter in PCa cells were attenuated by TGF-β signaling inhibitor SD208 (1 μM). Error bars represent the mean ± S.D. of three independent experiments. *P < 0.05.

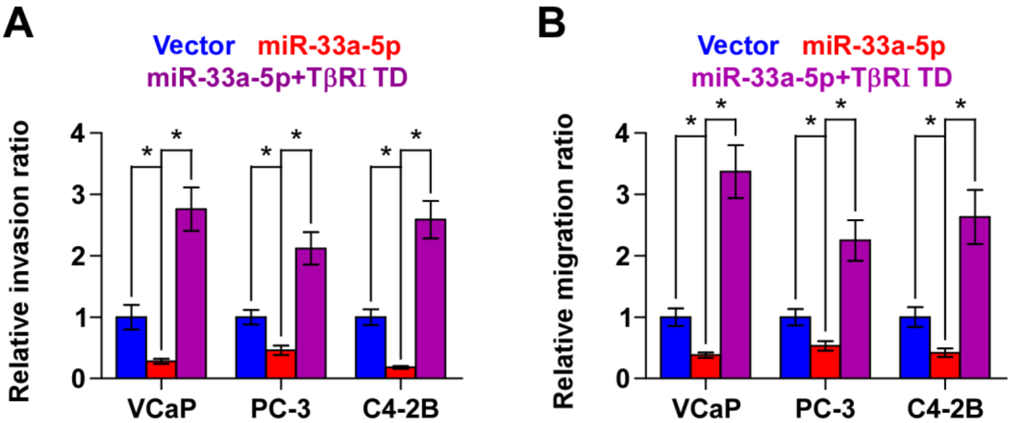

Figure S9. A and B, The inhibitory effects of miR-33a-5p overexpression on the invasion **(A)** and migration **(B)** abilities of PCa cells were attenuated by reconstitution of TGFBRI. Error bars represent the mean ± S.D. of three independent experiments. *P < 0.05.

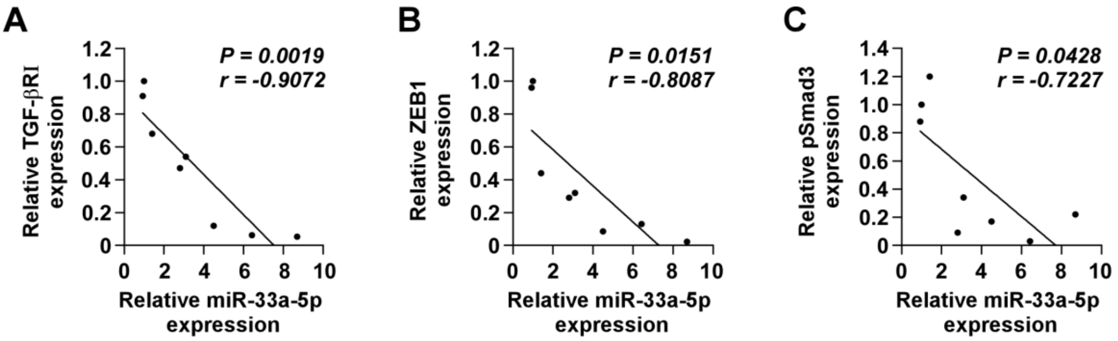

Figure S10. A-C, Correlation between miR-33a-5p levels and TGFBRI, ZEB1 and pSMAD3 expression in PCa tissues.The protein expression levels of TGFBRI, ZEB1 and pSMAD3 expression in PCa tissues were quantified by densitometry using Image J Software, and normalized to the levels of α-tubulin and p84, respectively. The sample 1 was used as a standard. The relative expressions of miR-33a-5p and these proteins were used to perform the correlation analysis.
